# High-Fat Diet in Perinatal Period Promotes Liver Steatosis and Low Desaturation Capacity of Polyunsaturated Fatty Acids in Dams: A Link with Anxiety-Like Behavior in Rats

**DOI:** 10.3390/nu17071180

**Published:** 2025-03-28

**Authors:** Lorena Mercado-López, Yasna Muñoz, Camila Farias, María Paz Beyer, Robinson Carrasco-Gutiérrez, Angie Vanessa Caicedo-Paz, Alexies Dagnino-Subiabre, Alejandra Espinosa, Rodrigo Valenzuela

**Affiliations:** 1 Department of Nutrition, Faculty of Medicine, University of Chile, Santiago 8380453, Chile; lorena.mercado@gmail.com (L.M.-L.); yasnamunoz.nut@gmail.com (Y.M.); camila.farias.c@uchile.cl (C.F.); m.pazbeyer@gmail.com (M.P.B.); 2 Direccion de Postgrado, Facultad Medicina, Universidad Andres Bello, Santiago 8370035, Chile; 3 Escuela de Nutrición y Dietética, Facultad de Farmacia, Universidad de Valparaíso, Valparaíso 2360102, Chile; 4 Laboratory of Stress Neurobiology, Interdisciplinary Centre for Health Studies (CIESAL), Institute of Physiology, Universidad de Valparaíso, Valparaíso 2360102, Chile; robinson.carrasco@gmail.com; 5 Escuela de Agronomía, Facultad de Ciencias Agronómicas y de los Alimentos, Pontificia Universidad Católica de Valparaíso, Quillota 2260000, Chile; angie.caicedo@udea.edu.co; 6 Escuela de Medicina, Campus San Felipe, Universidad de Valparaíso, San Felipe 2170000, Chile; ealejand@uchile.cl; 7 Department of Medical Technology, Faculty of Medicine, University of Chile, Santiago 8380000, Chile

**Keywords:** *n*-3 polyunsaturated fatty acid, fatty acid metabolism, alpha-linolenic acid, linoleic acid, docosahexaenoic acid, high-fat diet, anxiety, pregnancy

## Abstract

Background/Objectives: This study investigates the effects of a high-fat diet (HFD) during pregnancy and lactation on maternal and offspring health, focusing on behavioral, metabolic, and fatty acid composition outcomes in a rat model. Methods: Twelve female Sprague–Dawley rats were fed either a control diet, CD (n = 6), or HFD (n = 6) for 12 weeks, encompassing mating, gestation, and lactation periods (18 weeks). Anxiety-like behavior, maternal behavior, depression-like behavior, and social play were studied. Post mortem, the liver function, hepatic steatosis, and fatty acid composition (erythrocytes, liver, adipose tissue) were evaluated. In regard to desaturase enzymes (Δ-6D and Δ-5D), liver activity, protein mass, and gene expression (RT-PCR) were analyzed. Additionally, gene expression of PPAR-α, ACOX, CPT1-α, SREBP-1c, ACC, and FAS was assessed. Statistical analysis was performed using Student’s *t*-test, mean ± SD (*p* < 0.05). Results: The HFD significantly increased maternal weight and anxiety-like behavior while reducing social interactions exclusively in male offspring (*p* < 0.05). It also led to a significant decrease in the synthesis and content of *n*-3 PUFAs in the analyzed tissues, induced hepatic steatosis, and upregulated the expression of pro-lipogenic genes in the maternal liver. Conclusions: These findings suggest that long-term HFD consumption alters tissue fatty acid composition, disrupts metabolic homeostasis, and contributes to behavioral changes, increasing anxiety-like behaviors in pregnant dams and reducing social interactions in male offspring. Overall, this study provides further insight into the detrimental effects of HFD consumption during the perinatal period.

## 1. Introduction

An adequate intake of both energy and nutrients are critical in women during pre- and post-pregnancy and breastfeeding periods because it has been demonstrated that maternal nutritional status is fundamental for the offspring growth and development [1]. Indeed, according to the Developmental Origins of Health and Disease (DOHaD) hypothesis, the etiology of several disorders manifesting in childhood and adulthood can be traced back to the prenatal and early infant phases as an extremely sensitive time window for nutritional programming [2,3]. In this regard, women with obesity exhibit a pro-inflammatory state and elevated oxidative stress parameters; this altered metabolic state during pregnancy has short- and long-term consequences for multiple fetal and neonate organs [4,5]. Hence, the offspring of obese mothers have an increased risk of developing metabolic diseases such as obesity or type 2 diabetes, as well as neuropsychiatric disorders, asthma, atopy, and cancer [1,4].

Moreover, in maternal obesity, the excessive gestational weight gain affects the fatty acid (FA) supply to the fetus and neonate, altering both placental FA transfer and breast milk FA composition during lactation [6]. Specifically, under this pathological condition, a change in the polyunsaturated FA (PUFA) profile has been observed, with lowering the *n*-3 and raising *n*-6 PUFA levels in both maternal and fetal circulation during pregnancy, and also in the breast milk [2,3]. An optimal *n*-3:*n*-6 PUFA ratio is crucial for adequate physiological functions in cells, tissues, and organs. FAs in general and PUFA in particular exhibit a key role in the fetus and newborn neurodevelopment [7]. In fact, the central nervous system contains the second highest concentration of lipids in the organism after the adipose tissue (50–60% of the brain’s dry weight) [8]. Within the PUFA fraction, the *n*-6 and *n*-3 families are the most physiologically significant in terms of both quantity and function [7]. The main representatives of each family are arachidonic acid (ARA, 20:4*n*-6) for *n*-6 family with ~50% of the brain’s PUFA content and docosahexaenoic acid (DHA, C22:6*n*-3) for *n*-3 family with ~45% [9]. In humans, DHA could be acquired directly from the diet or could be synthesized from alpha-linolenic acid (ALA, C18:3*n*-3) by the liver [10]. In the same way, ARA is either ingested or produced from linoleic acid (LA, C18:2*n*-6) [7], since ARA is widely distributed in different foods and is always covered through diet [4]. Unfortunately, DHA or its precursor ALA is infrequently consumed in sufficient quantity. Animal models show that *n*-3 PUFA deficiency leads to low levels of DHA in the offspring’s cerebral cortex, affecting learning ability, which is also related to a higher prevalence of mood disorders [9,11].

An adequate supply of *n*-3 PUFAs is important for developing a healthy pregnancy; specifically, maternal *n*-3 PUFA supplementation can increase offspring birth weight and decrease preterm risk, prolonging gestational duration [3]. Moreover, it positively impacts maternal health by reducing the incidence of preeclampsia [11].

Obesity during pregnancy significantly impacts both maternal and offspring health [1,12]. Maternal obesity is associated with a heightened risk of gestational diabetes, hypertensive disorders, and complications during labor and delivery [4,12]. For the offspring, maternal obesity can lead to adverse outcomes such as macrosomia, preterm birth, and an increased likelihood of developing obesity and metabolic disorders later in life [5,11]. Additionally, maternal obesity can impair placental function, affecting nutrient transfer; more specifically, altered PUFA transfer in obese pregnant women has been documented [6]. Moreover, *n*-3 PUFAs have anti-inflammatory properties that can mitigate some of the adverse effects of obesity [5]. However, obesity can alter the metabolism and efficacy of *n*-3 PUFAs, potentially diminishing their beneficial effects [10]. Obese individuals often exhibit higher levels of oxidative stress in the liver and a consequent reduction in the activity of desaturases enzymes, specifically Δ-5 desaturase (Δ-5 D) and Δ6-D (Δ-6 D) enzymes. This results in a lower level of PUFAs, specially DHA [10,13], an effect that may be relevant in the perinatal period, as discussed earlier.

Finally, recent studies suggest an association between *n*-3 PUFA levels and the prevalence of mood disorder [7,8]. This association has been observed in the general population and also in the perinatal stage [9], suggesting that an insufficient supply of DHA (or precursors) could result in a high risk to develop anxiety and depression [14]. Consequently, understanding and addressing the interactions among obesity, *n*-3 PUFAs metabolism, and their implications on maternal and offspring behavior are critical for developing effective nutritional and therapeutic strategies. According to this background, the aim of this study was to assess the effect of a long-term high-fat diet (HFD) consumption continuing through perinatal (pregnancy and breastfeeding) periods on the Sprague–Dawley dams and its offspring. The study examined liver parameters associated with steatosis and lipid metabolism. In this regard, special focus has been deposited on the expression and activity of hepatic Δ-5 D and Δ6-D enzymes and the levels of *n*-6 and *n*-3 PUFAs and their relation with dams and offspring behavior. The study hypothesis is that long-term maternal consumption of an HFD continuing through gestation and lactation impairs maternal DHA synthesis, leading to reduced accumulation in the offspring’s brain and alterations in anxiety-like behavior in Sprague–Dawley rats.

## 2. Materials and Methods

### 2.1. Animals and Experimental Design

Animals: Twelve eight-week-old female rats of the Sprague–Dawley strain were obtained from the central animal facility of the Pontificia Universidad Católica de Chile. The rats were housed in the animal facility of the Laboratory of Stress Neurobiology at Universidad de Valparaíso on a standard schedule of 12 h of light and 12 h of darkness (lights were turned on at 07:00 a.m.). Food and water were available ad libitum. All animal procedures were approved by the Bioethics Committee for Research in Animals of the Faculty of Sciences of Valparaiso University (BEA182-22). The sample size was calculated using the statistical software G*Power 3.1. The critical variable considered was the concentration of DHA in the brain (20% reduction consuming an HFD), with a statistical power of 80% and a significance of 0.05 according to previous publications of our group [13].

**Figure 1:**
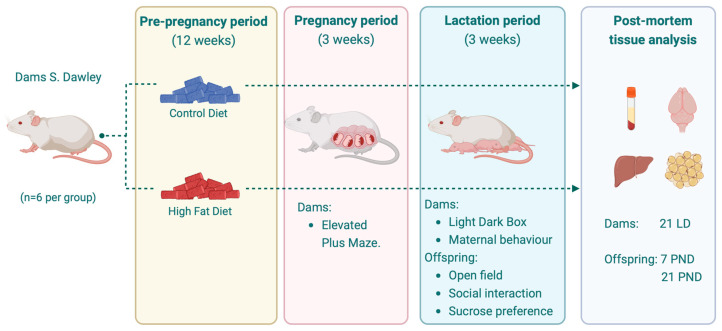
Experimental design. PND = post-natal day, LD = lactation day. Created with BioRender.com.

Experimental design: *S. Dawley* females were randomly assigned to the different dietary intervention groups for 12 weeks with control diet (CD, n = 6) with 2% ALA (D22072209, Research diet, New Brunswick, NJ, USA) and high-fat diet (HFD, n = 6) with 2% ALA (D22072206, Research diet, New Jersey, NJ, USA). Diet composition is available in Supplementary Table S1. After mating, all pregnant rats were placed in individual cages with environmental enrichment material to avoid gestational stress. The offspring were nursed by their mothers until postnatal day 21 (PND21). One dam from the HFD group presented dystocia during delivery and was euthanized to avoid pain and suffering. Once the lactation period was over, the pups were separated from their mothers and received the same diet assigned to the mother. The dam’s anxiety-like behavior was evaluated at 19th gestational day with Elevated Plus Maze (EPM) and Light Dark Box (LDB) at 4th postpartum day. Maternal behavior was assessed at 4 PND, and between 4 and 14 PND, experiments were performed to evaluate reflexes related to neurodevelopment. In pups, open-field test (OFT), social interaction, and sucrose preference test (SPT) were applied at PND 24–PND 27 to evaluate anxiety-like behavior [15,16]. For post mortem analysis, at PND7 and 21, two pups were randomly selected, one male and one female from each litter, and anesthetized with isoflurane and euthanized. Blood samples were obtained by cardiac puncture for metabolic serum parameter assessment. The rats were transcardially perfused with saline (0.9% NaCl), and tissues were collected. Liver, adipose tissue (retroperitoneal fat, inguinal fat, and mesenteric fat), and brain (only from offspring) were frozen in liquid nitrogen and stored at −80 °C for specific determinations detailed below. At PND21, the respective mother was included in the euthanasia process (Figure 1).

### 2.2. Serum Parameters

Serum glucose was quantified using a glycemia kit (Wiener lab, Santa Fe, Argentina) and a clinical photometer (BioSystems, BTS 350, Barcelona, Spain). Insulin was quantified using an ultrasensitive insulin ELISA kit (Mercodia, Uppsala, Sweden), and the optical density of the wells of each plate was read in the plate reader (Bio-Rad^®^ 550 ELISA, Hercules, CA, USA). Insulin resistance was estimated by the homeostasis model assessment method (HOMA) [fasting insulin (μU/mL) × fasting glucose (mM)/22.5] [17]. Regarding the serum lipid profile, triglyceride (TG) levels were determined using specific kits (Wiener Lab, Santa Fe, Argentina) and a clinical photometer (BioSystems, BTS 350). Total cholesterol (T-Cho) and HDL-cholesterol (HDL-c) levels were measured using test strips (KENSHIN-2, Shiga-ken, Japan) and a dry chemistry analyzer (SPOTCHEM EZ SP-4430, Kyoto, Japan). In terms of serum levels of transaminases, Gamma-glutamil transferase (GGT), Glutamat–Pyruvat-Transaminase (GPT), and Glutamic oxaloacetic transaminase (GOT) were measured using reagent strips (KENSHIN-2, Lot PJ2A76) and a dry chemistry analyzer (SPOTCHEM EZ SP-4430, Kyoto, Japan).

### 2.3. Liver Parameters

Histological evaluation: Liver samples were fixed in phosphate-buffered formalin, embedded in paraffin, stained with haematoxylin–eosin, and analyzed by optical microscopy in a blind fashion to evaluate the hepatic steatosis score according to Brunt et al. [18]. This index is graded as 0 (absence of hepatocytes infiltrated with fat), 1 (mild, 5% to 34% of liver cells with fat), 2 (moderated, 35% to 65% of infiltrate hepatocytes) and 3 (severe, >65%).

Quantification of the protein concentration of Δ-6D, Δ-5D in dam’s liver: Assessment of Δ-6D and Δ-5D protein concentration (ng/g tissue) of each enzyme in liver was carried out using commercially available enzyme-linked immunosorbent assay kits (MyBioSource, Inc., San Diego, CA, USA), according to the manufacturer’s instructions. Briefly, samples were prepared as indicated by the manufacturer’s instructions, and plates were read at 450 nm. The protein concentration was quantified in each tissue (ng/g tissue) using a standard curve [13].

Desaturases Δ-6D, Δ-5D activity in dam’s liver: Liver samples (500 mg) previously stored at −80 °C were homogenized on ice in a buffer solution of pH 7.9 containing 10 mmol/L HEPES, 1.0 mmol/L EDTA, 0.6% Nonidet P-40, 150 mmol/L NaCl, and protease inhibitors (1 mmol/L phenylmethylsulfonyl fluoride, 1 μg/mL aprotinin, 1 μg/mL leupeptin, and 1 mmol/L orthovanadate). Tissue homogenates were centrifuged at 4 °C, first at 2000× *g* for 30 s, followed by centrifugation of the supernatants at 5000× *g* for 5 min, and finally at 100,000× *g* for 60 min, to obtain the microsomal isolations for the assessment of desaturase activities. These were assayed using 1 mL of incubation medium containing 4 μmol ATP, 0.1 μmol coenzyme-A, 1.28 μmol NADPH, 2.42 μmol N-acetylcysteine, 0.5 μmol nicotinamide, 5 μmol MgCl_2_, 62.5 μmol NaF, and 62.5 μmol phosphate buffer pH 7, with addition of 100 nmol albumin-bound FA precursor and 1 mg protein of cytosolic extract in a total volume of 100 μL. The specific enzymatic reactions to determine the activity for *n*-3 or *n*-6 PUFAs were the following: (i) Δ-6D activity was determined by the amount of ALA converted to stearidonic acid (SDA, C18:4*n*-3) and (ii) Δ-5D activity was determined by the amount of dihomo–Gamma–linolenic acid, (DGLA, C20:3*n*-6) converted to ARA, according to Valenzuela et al. [13].

### 2.4. Analysis of Fatty Acid by Gas Chromatography

The extraction of total hepatic, adipose tissue, brain (offspring) tissues, and erythrocytes was carried out according to Bligh and Dyer [19]; pulverized livers (~100 mg per sample) were homogenized with a chloroform/methanol mixture (2:1 *v*/*v*) in a cold environment. Distilled water and 0.5 N MgCl_2_ were added to the homogenized product and centrifuged at 0–4 °C (3500 rpm × 5 min) to collect the chloroform phase. Samples were then dried under a stream of nitrogen, and fatty acid methyl esters (FAMEs) were prepared according to the technique described by Morrison and Smith [20]. The saponifiable lipids obtained from liver tissue were prepared with boron trifluoride (BF_3_, 12% methanolic solution) followed by treatment with NaOH saturated in methanol (0.5 N). Finally, FA were extracted and collected to be quantified by gas–liquid chromatography (GC) in an Agilent Hewlett–Packard equipment (model 7890B serial CN13523084, Santa Clara, CA, USA) using a capillary column (Agilent HP-5 30 m × 320 μm × 0.25 μm) and a flame ionization detector. The FAs were identified by comparing their retention times and using C23:0 fatty acid as an internal standard (Nu-Chek Prep Inc., Elysian, MN, USA). The results were expressed as percentage moles of FAs.

### 2.5. Gene Expression Assays

Total RNA was isolated from dams and offspring liver samples using Trizol (Ambion, Carlsbad, CA, USA), according to the supplier’s protocols. Purified RNA was treated with DNAase I (DNA-freeTM Kit; Invitrogen, Vilnus, Lithuania). Then, 1 µg of total RNA was used to generate single-stranded cDNA (PPAR-α: Peroxisome proliferator-activated receptor alpha, ACOX: Acyl-CoA oxidase, CPT1-α: Carnitine palmitoyl transferase 1α, SREBP-1c: Sterol response element binding protein, ACC: Acetyl-CoA-carboxylase, FAS: FA synthase; FADS-2 codifies for Δ-6D and FADS-1 codifies for *Δ-5D* using High-Capacity cDNA Reverse Transcription Kit (Applied biosystems, Vilnus, Lithuania), but only for dam’s liver samples assessed mRNA of Δ-6D) and Δ-5D. The cDNA was amplified using the TaqMan^®^ Gene Expression Assays (Applied biosystems, Pleasanton, CA, USA) in a total volume of 20 µL. Real-time PCR was performed in an AriaMx qPCR System (Agilent Technologies, Penang, Malaysia) following the manufacturer’s recommendations (Applied Biosystems, Pleasanton, CA, USA). Expression levels of target genes studied were normalized by the expression of glyceraldehyde 3-phosphate dehydrogenase (GAPDH) as internal control. Fold change between groups was calculated by the 2−(ΔΔCt) method, as established by Pfaffl [21] (gene-specific TacMan probes were used in the study; see Supplementary Table S2).

### 2.6. Behavioral Procedures

Tests were performed between 8:00 a.m. and 11:00 a.m. (excepting maternal behavior) on naive rats recorded by cameras connected to a computer in an adjacent room. Videos were analyzed by EthoVision^®^ Software XT 18 version (Noldus, Wageningen, The Netherlands). Mazes were cleaned with 10% ethanol solution between trials. Animals from all experimental groups were evaluated simultaneously in a sound-proof and temperature-controlled (21 ± 1 °C) room. The background noise level in the room was 40 dB (precision sound level meter, Model #1100 Quest Technologies, Medley, FL, USA).

#### 2.6.1. Dam’s Anxiety-Like Behaviors

Elevated plus maze (EPM): This test evaluates anxiety based on rodents’ aversion to open and elevated spaces. Increased time spent in closed arms indicates heightened anxiety levels. The EPM is widely used due to its sensitivity and reliability in detecting anxiety-like behaviors [15,22]. The elevated plus maze consisted of two open (60 × 15 cm) and two closed (60 × 15 × 20 cm) arms arranged opposite each other extending from a central platform (15 × 15 cm). It was elevated 100 cm and illuminated by a ceiling bulb, providing 300 ± 10 lux in open arms and 210 ± 10 lux in closed arms. Each rat on the 19th day of pregnancy was placed at the center of the maze, always facing the same open arm, and the frequency of entries into the open and closed arms during a 5 min test period was recorded. Entries into an arm were defined as occurring when the animal placed 70% of its body onto the arm. Open-arm entries were used as a measure of anxiety level [15,22].

Light Dark Box (LDB) paradigm: This assay exploits rodents’ preference for dark, enclosed spaces over illuminated areas. The amount of time spent in the light compartment versus the dark compartment serves as an index of anxiety-like behavior. The LDB is a standard tool for assessing anxiety responses in rodents [16]. In order to quantify anxiety-like behavior in dams on the 4th postpartum day with a different behavioral test, the LDB paradigm was used. The LDB consisted of a two-compartment Plexiglass box, each measuring 50 cm × 50 cm × 40 cm. The lighted chamber was illuminated from above by a white light bulb (500 lux at floor level), while the dark chamber was made with black Plexiglas with a light intensity of 5 lux on the floor. Both chambers were separated by a black partition with a small opening (8 cm × 8 cm) at the bottom. Each rat was placed in the center of the lighted box, facing away from the door, and released during the 5 min test.

#### 2.6.2. Maternal Behavior’s Assesment

Evaluation of maternal behaviors, such as nursing, grooming, and pup retrieval, provides insights into the dams’ affective states. Alterations in these behaviors can be indicative of anxiety or stress, which may subsequently influence offspring development [23]. In this study, they were assessed with two complementary procedures. In the first procedure, CD and HFD dams were evaluated in their home cages on lactation day 2. Behavior was monitored every 4 min over a 60 min observation period every second hour between 08.00 h and 21.00 h [23]. Behaviors were classified as self-directed (outside the nest, drinking water, eating, and self-grooming) and pup-directed (arched back and non-arched back postures, blanket position, licking, and grooming). The second procedure was the pup retrieval test. It was applied on PND3, according to a previously described protocol [24]. The dam was separated from the litter for less than 3 min and was kept in a holding cage. Meanwhile, the pups were placed in the corner of the cage opposite the nest. Then, the dam was returned to the cage. The behaviors scored were the following: (i) latency to first pup-contact (latency to sniff or contact the first pup), (ii) total time to retrieve pups back to the nest, (iii) the percentage of the time that dams spent performing maternal behaviors (nest building and transporting), and (iv) other behaviors not directed to the pups (rearing and self-grooming). The test ended after 10 min, or when the female had retrieved all the pups. At the end of the test, the observer returned the pups that their mother did not pick up to the nest site.

#### 2.6.3. Offspring Behavior’s Assesment

Social interaction (SI): Male and female rats (PND24) from the CD and HFD groups were tested for spontaneous social interaction. This paradigm evaluates the propensity for social engagement, where reduced interaction time may indicate increased anxiety or social withdrawal. The SI test is instrumental in assessing social behaviors and anxiety-related responses [25,26]. We used a modified version of the protocol described by Malkesman and Weller [25]. The pups were kept individually for 12 h prior to testing. The test was conducted in a clear acrylic glass chamber (38 × 21 × 18 cm), the floor of which was covered with approximately 1 cm of pine shavings. After 12 h of isolation, each pup was recorded for 15 min in a cage with an unfamiliar conspecific pup (same breed, comparable age and sex, but from a different litter). The tests were conducted under infrared light. The frequency of behaviors was analyzed from the videotapes. Every time a pup started some specific behaviors, like exploratory sniffing, anogenital sniffing, social grooming, and social play (e.g., boxing, chasing, pinning, attacking, etc.), this was scored as “spontaneous social interaction” [26].

Open field test: This test measures exploratory behavior and general activity levels in a novel environment. Increased time spent in the periphery and reduced center exploration are interpreted as anxiety-like behaviors. The OFT is a widely used assay for assessing anxiety and locomotor activity [27]. Rats were placed individually in the center of a black Plexiglass cage (40 × 40 × 40 cm) for 5 min. The arena was illuminated to 300 lux (measured using a digital lux meter; Model # LX-1010B; Weafo Instrument Co., Shanghai, China). Total distance traveled, mean speed, and the time that rats spent in the perimeter (anxiety-like behavior marker) were automatically analyzed from video recordings. Heat maps of representative rat tracking from each experimental group were analyzed using the Noldus behavioral tracking system. In all experiments, animals from the CD and HFD groups were evaluated at the same time.

Sucrose preference test: This test assesses anhedonia by measuring the preference for a sweet solution over water. A decreased preference is indicative of reduced sensitivity to rewarding stimuli, often associated with depressive-like states. The SPT is a standard measure for evaluating anhedonia in rodents [25,27]. The rats were first trained for 3 days to drink sweet liquid (5% sucrose) as previously published by Bravo-Tobar et al. [27]. Then, animals were water-deprived for 12 h before the test. During the test, the rats were allowed to choose between two bottles for 12 h, one containing a 5% sucrose solution and the other containing only water. The amount of liquid consumed by the rats was measured, and the percentage of preference of sucrose solution in relation to the neutral liquid was calculated.

### 2.7. Statistical Analysis

The Shapiro–Wilk test was applied to verify the normality distribution. Main effects and interactions between diets were analyzed with Unpaired *t*-test with Welch’s correction. Two-way ANOVA was used to analyze the effect between sex and diet with Sidák post-test. All statistical analysis was conducted using Graphpad Prism 8 (GraphPad Software, Inc., Boston, MA, USA). The results were expressed as mean values with their standard deviation. The results were considered statistically significant at *p* < 0.05.

## 3. Results

### 3.1. Evolution of Body Weight and Food Intake in Dam’s Rats

**Figure 2:**
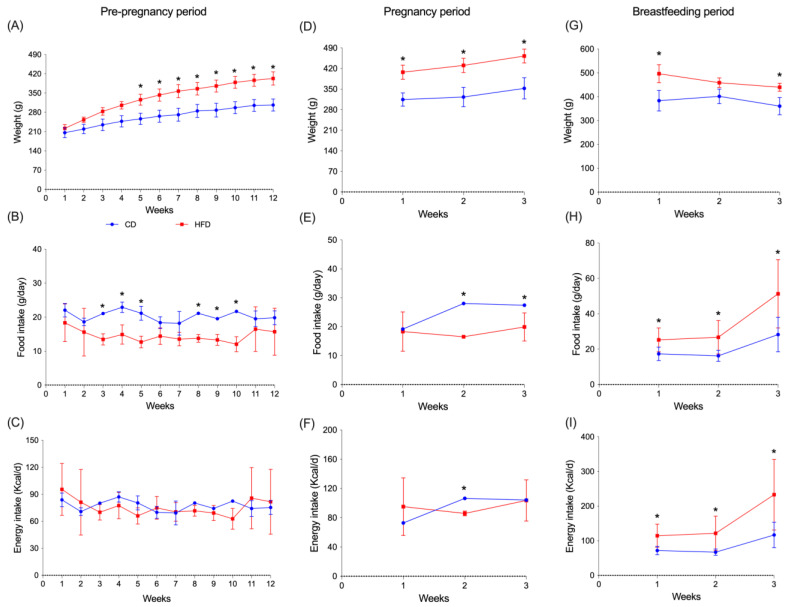
Maternal body weight gains and food intake in g/day and kcal/day during pre-pregnancy (**A**–**C**), pregnancy (**D**–**F**), and the postnatal period (**G**–**I**) in dams fed with control diet (CD), high-fat diet (HFD). Data are expressed as mean ± DS. n = 12. CD, control diet (n = 6); HFD, high-fat diet (n = 6). Significant differences between groups are indicated with *. Differences were calculated by two-way ANOVA followed by Sidák (*p* < 0.05) between CD (n = 6) and HFD (n = 6).

Figure 2 shows the effects of diets on maternal weight gain, energy, and food intake. Adipose tissue and liver weight were also assessed. Figure 2A shows that weight gain was significantly higher in the HFD group from week five (*p* = 0.020), six (*p* = 0.018), seven (*p* = 0.014), eight (*p* = 0.020), nine (*p* = 0.016), ten (*p* = 0.006), eleven (*p* = 0.006) to twelve (*p* = 0.005) compared to the CD group. This situation was maintained throughout the pregnancy period (Figure 2D) showing a significant increment in all the three weeks (*p* = 0.001; 0.006; 0.009), respectively. Likewise, during the lactation period (Figure 2G), weight was increased in the HFD group in the first week (*p* = 0.018) and third week (*p* = 0.046) compared to the CD group. Figure 2B shows a significant increase in the intake of the diet (g/day) in the CD group.

The increase was observed during the period prior to pregnancy, in weeks three, four, and five (*p* = 0.004; 0.015; 0.007) and weeks eight, nine, and ten (*p* < 0.001: *p* = 0.011; 0.007) compared to HFD. However, this increase was not reflected in a significant increment of energy intake (kcal/day) (Figure 2C). During the pregnancy period (Figure 2E), an increase in the CD group versus HFD group was observed at weeks two and three (*p* < 0.001; *p* = 0.036), respectively. Finally, during the lactation period (Figure 2H), an increase in the HFD group compared to the CD groups was observed in all weeks (*p* = 0.031; 0.030; 0.024). Figure 2F shows caloric intake during pregnancy; it was higher in the HFD-fed group compared to the CD group in the second week of pregnancy (*p* < 0.001), and during the lactation period (Figure 2I) a higher caloric intake was observed in the HFD group in the first, second, and third week (*p* = 0.025; 0.049; 0.043), respectively, compared to the CD group.

### 3.2. Body Weight in Offspring Rats

**Figure 3:**
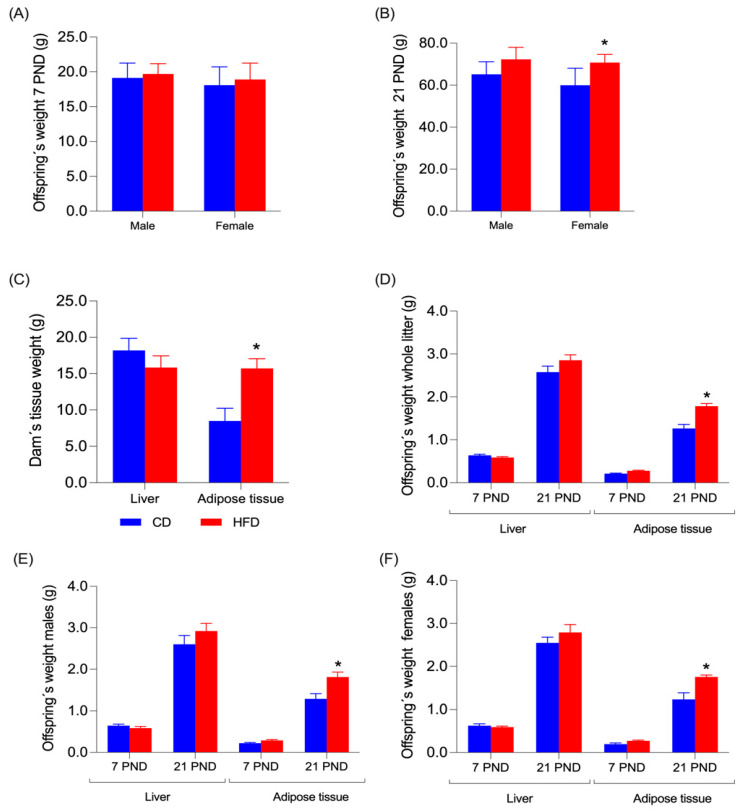
Adipose tissue and liver weights in mothers. (**A**) Offspring weight on PND7. (**B**) Offspring weight on PND21. (**C**) Dam’s tissue weight. (**D**) Tissue weights of whole litter offspring at PND7 and 21. (**E**) Male tissue weight at PND7 and 21. (**F**) Female tissue weight at PND7 and 21. Data are expressed as mean ± SD. Significant differences between groups are indicated with *. Differences were calculated by ANOVA two-way followed by Sidák (*p* < 0.05) between CD (n = 6) and HFD (n = 6).

Two randomly selected offspring, one male and one female from each litter, were euthanized at 7 and 21 days after birth (PND7 and PND21). Before the procedure, the offspring were weighed, and the data are presented in Figure 3A,B for PND7 and PND21, respectively. No significant differences were found between offspring fed a CD and those fed an HFD, or between males and females at PND7. At PND21, no differences in weight were found between males fed a CD or a HFD, but females fed with a HFD weighed more than females fed with a CD (*p* = 0.0304) (Figure 3B).

### 3.3. Liver and Adipose Tissue Weight in Dams and Offspring

After delivery, the weights of the dam tissues and the weight of the pups were evaluated; that information is shown in Figure 3. In the dams (Figure 3C), no significant difference was observed between liver weight among groups. But an increase in adipose tissue (comprising retroperitoneal, inguinal, and mesenteric fat) was observed in the HFD group compared to the control group (*p* = 0.088). On postnatal day 7, no significant differences were observed in liver and adipose tissue in both sexes (Figure 3D–F). Nonetheless, in the whole litter (female and male offspring), a greater weight of adipose tissue was observed on postnatal day 21 (Figure 3D) in the HFD group versus the control (*p* < 0.001). The same situation was observed in male pups alone (Figure 3E) on day 21 of the HFD group (*p* = 0.035) and female pups (Figure 3F) on day 21 of the same group (*p* = 0.004) compared to the control group.

### 3.4. Dam and Offspring Serum and Liver Parameters

Table 1 shows metabolic and liver parameters from dams. No significant differences were found between CD and HFD in glycemia, HOMA-IR, TG, T-Cho, HDL-c, GGT, GPT, and GOT. However, the insulin value was significantly higher in dams fed with HFD compared to the CD group. Table 2 displays metabolic and liver parameters from offspring obtained at 7 and 21 postnatal days. In glycemia, insulin, HOMA-IR, T-Cho, HDL-c, and GOT, there were no significant differences observed. The TG value was significantly higher in HFD at both PND7 (*p* = 0.058) and PND21 (*p* = 0.0493). GPT was only significantly higher in HFD-group at PND21 (*p* = 0.0288), but GGT was significantly higher at PND7 (*p* = 0.0422).

**Figure 4:**
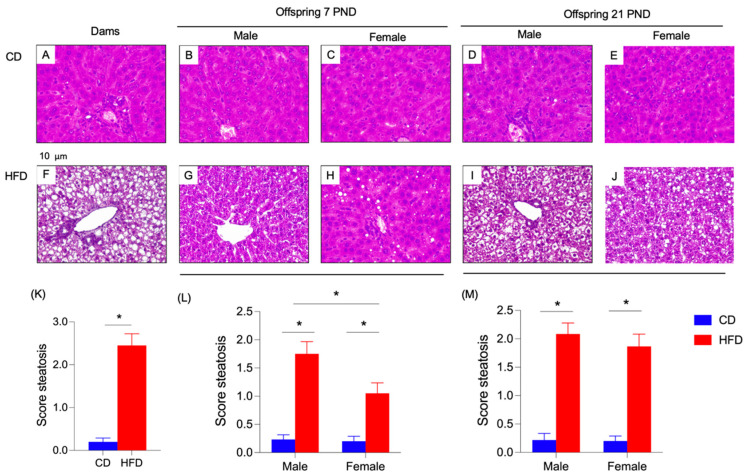
Morphological characteristics in the liver of dams and offspring. Representative liver hematoxylin and eosin staining. Rats subjected to CD and HFD. Representative liver sections from a total of 6 animals per experimental group (hematoxylin–eosin; original magnification 10×). (**A**–**E**) images of Dams and offspring with CD, (**F**–**J**) images of Dams and offspring with HFD, (**K**) Score of steatosis in Dams, (**L**) score of steatosis at 7 PND, (**M**) score of steatosis at 21 PND. Significant differences between groups are indicated with *. Differences were calculated by ANOVA two-way followed by Sidák (*p* < 0.05) between CD (n = 6) and HFD (n = 6).

Figure 4 shows the liver histology from dams and offspring obtained from different times of intervention. The animals subjected to CD exhibited a normal liver histology (Figure 4A–E) compared to the rats that received HFD (Figure 4F–J). In this regard, dam’s steatosis score showed a significant high value in the HFD group (Figure 4K). In both male and female and at PND7 and 21, HFD generated an increment in steatosis score (Figure 4L,M). Also, in male offspring at PND7, a significant rise in liver steatosis score was shown with respect to the female rats (Figure 4L). But on PND21, no significant differences were detected between male and female specimens (Figure 4M).

### 3.5. Fatty Acid Profile from Different Tissues in Dams and Offspring

As previously detailed (Figure 1), samples from dams and offspring of liver, brain, adipose tissue (retroperitoneal fat, inguinal fat, and mesenteric fat), and erythrocytes were collected, and then analyzed by gas chromatography to characterize FA composition. Results are detailed in Table 3 (dam liver, adipose tissue, and erythrocytes), Table 4 (offspring liver), Table 5 (offspring adipose tissue), Table 6 (offspring erythrocytes) and Table 7 (offspring brain). The FA composition of pup stomach content (indirect measure of maternal milk) at 7 days is available in Supplementary Table S3.

Table 3 shows the FA composition in liver, adipose tissue, and erythrocytes samples from dams at three weeks postpartum. In the liver, the total saturated fatty acid (SFA) and miristic acid (C14:0, MA) levels were not significantly different between the CD- and the HFD-fed groups. Palmitic acid (C16:0, PA) was significantly higher in the CD group with respect to the HFD group. However, stearic acid (C18:0, SA) was significantly higher in the HFD group compared to the CD group. Total monounsaturated FA (MUFA) and palmitoleic (C16:1, POA) and oleic acids (C18:1*n*-9, OA) were significantly higher in the CD group with respect to the HFD group. The total PUFA, total *n*-6 PUFA, total *n*-3 PUFA, LA, ALA, AA, eicosapentaenoic acid (EPA), DHA, and the *n*-6:*n*-3 ratio were significantly lesser in the CD group with respect to the HFD-fed group. N-6 docosapentaenoic (C22:5*n*-6, *n*-6 DPA) and *n*-3 docosapentaenoic (C22:5*n*-3, *n*-3 DPA) did not have significant differences. Total SFA and MA in adipose tissue significantly increased in HFD compared to CD, even though PA was diminished in HFD. Also, a lower percentage of mmol was found in total MUFA (*p* = 0.005) and POA in HFD-fed group. In PUFA, almost all FA presented a significant rise with HFD, both in the *n*-3 PUFA (ALA, EPA, DHA) and *n*-6 PUFA (LA, AA). In consequence, DPA-*n*-6, OA, or SA and the ratio of *n*-6:*n*-3 were not significantly different between diets. In erythrocytes, a different situation was observed in SFA. MA and PA presented lower levels in HFD while SA increased significantly (*p* = 0.005). Total SFA showed no differences between groups. In contrast, all MUFAs, including POA, OA, and total MUFA, had a significant reduction in HFD. While total PUFA showed an increase (*p* < 0.001), the ratio of *n*-6:*n*-3 show no differences. An increase was observed in LA (*p* < 0.001) and DHA *p* = 0.008), meanwhile a decrease in ALA (*p* = 0.001) and DPA-*n*-6 (*p* = 0.025) was found. In DPA-*n*-3, EPA, and ARA, no differences were observed.

Table 4 displays the results of FA composition in the liver from male and female offspring at PND7 and PND21. SFA and PA exhibited a significantly higher content in the CD group in males and females, both PND7 and PND21, compared to the HFD group. MA and SA were considerably higher in females on PND7 and PND21 in CD compared to the HFD group. Total MUFA in males and females was significantly higher in PND21 in the CD group compared to the HFD group. POA was considerably higher in male and female PND7 and PND21 from the CD group compared to the HFD group. OA was significantly higher in males on PND21 in the CD group than in the HFD group. In the PUFA group, LA showed consistent significant differences between CD and HFD across all groups, with higher levels in the HFD group. AA showed substantial differences in males at PND7 and PND21. Total SFA and PUFA showed significant differences; in SFA, higher levels were found with CD, while in PUFA, higher levels were found with HFD. The ratio of *n*-6:*n*-3 shows a decrease in HFD females at PND7, but a significant increase in both males and females was found at PND21 with HFD.

Table 5 presents the results of fatty acid analysis in adipose tissue in male and female offspring at PND7 and PND21. SFA shows significant differences in MA and PA are observed across all groups, with generally higher levels in the CD group at PND7 and PND21 for both males and females. SA shows significant differences at PND21 in males and females, with higher levels in the HFD group. In MUFA, OA shows substantial differences at PND21 in males, with higher levels in the CD group and a trend towards significance in females. In PUFA, LA shows significant differences between diets, particularly with HFD showing significantly higher levels across all groups and days. ALA also shows substantial differences, particularly in females at PND21, where HFD substantially increases. Total SFA shows significant differences, with generally higher levels in the HFD group. Total PUFA shows notable increases in the HFD group across all comparisons; the ratio of *n*-6:*n*-3 shows an increase in HFD in males at PND7 and PND21. In females, a rise is observed only at PND21. FAs not mentioned show no differences between groups.

Table 6 provides data on the FA in erythrocytes from male and female offspring at PND7 and PND21. In SFA, PA shows significant differences between CD and HFD in males and females at PND21, with lower levels in the HFD group. SA also shows substantial differences, particularly in males at PND21 and females at PND7 and PND21, with HFD leading to higher levels. In MUFA, OA shows significant differences in males at PND7 and PND21, with higher levels in the CD group. A significant difference is observed only in females at PND21. In PUFA, LA shows substantial differences, particularly in males at PND7 and PND21, with higher levels in the HFD group. AA shows significant differences across multiple groups, especially at PND21 in males and females, with the HFD group having higher levels. Total PUFA significantly increases in the HFD group across all time points, especially at PND21. The ratio of *n*-6:*n*-3 shows significant differences, with the HFD group having a higher ratio at PND7 in both males and females. However, at PND21, it is only higher in females (*p* = 0.034). DPA*n*-6 and DPA*n*-3 show no differences in all sex and postnatal days.

The brain’s fatty acid composition of male and female offspring at PND7 and PND21 is presented in Table 7. Only a decrease (*p* < 0.001) in SFA is observed in MA at HFD males at PND7. In contrast, POA reduces HFD in all groups except females at PND21. OA levels are higher with HFD only in males at PND21. In PUFAs, LA is increased by HFD in males and females at PND7, but these values start to drop at PND21. ALA increases with HFD but only in males at PND7 and 21. AA generally has no variations, except in females at PND7, higher in the CD group. DPA*n*-6 shows lower levels of HFD in all groups. This situation is not observed for DPA*n*-3, which only diminishes females at PND7. EPA shows a slight male decrease at PND21, and DHA presents no significant differences. Finally, the ratio of *n*-6:*n*-3 is smaller with a HFD in males and females at PND21, but the values in females at PND7 are slightly higher.

### 3.6. Transcription, Quantitation, and Enzyme Activity in Liver from Dams and Offspring

**Figure 5:**
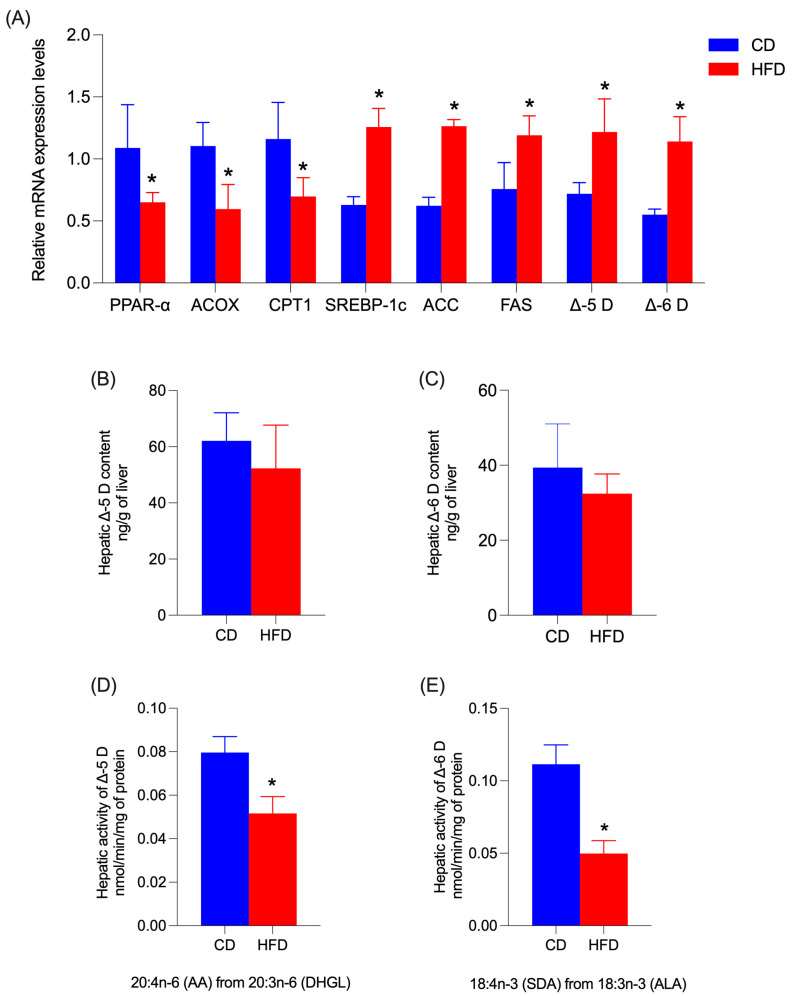
(**A**) Relative mRNA expression of peroxisome proliferator-activated receptor alpha (PPAR-α), Acyl-CoA oxidase (ACOX), carnitine palmitoyl transferase 1α (CPT1-α), sterol response element binding protein (SREBP-1c), Acetyl-CoA carboxylase (ACC), FA synthase (FAS). FADS-2 encodes Δ-6 Desaturase (Δ-6D) and FADS-1 encodes Δ-5 Desaturase (Δ-5D) in dam liver. (**B**,**C**) Quantification of Δ-6 Desaturase (Δ-6D) and Δ-5 Desaturase (Δ-5D) in dam liver. (**D**,**E**) Evaluation of Δ-6 Desaturase (Δ-6D) and Δ-5 Desaturase (Δ-5D) activity in dam liver. Data are expressed as mean ± SD. Significant differences between groups are indicated with *. Differences were calculated by ANOVA two-way followed by Sidák (*p* < 0.05) between CD (n = 6) and HFD (n = 6).

Figure 5 shows hepatic mRNA levels of PPAR-α, ACOX, CPT-1α, SREBP-1c, ACC, FAS, Δ-5D, and Δ-6D from dams. In dams that received a CD, a significant increment was observed in the mRNA levels of PPAR-α (*p* = 0.0312), ACOX (*p* = 0.0084), and CPT1-α (*p* = 0.0201) compared to the HFD group. Nonetheless, the dams fed with a HFD exhibited significant higher values for SREBP-1c (*p* = 0.0032), ACC (*p* = 0.0025), FAS (*p* = 0.0356), Δ-5D (*p* = 0.0025), and Δ-6D (*p* = 0.0015) with respect to the CD group (Figure 5A). There were no significant differences in the protein quantification of Δ-5D and Δ-6D between the two groups (Figure 5B,C). Despite these results, the enzymatic activity of Δ-5D and Δ-6D was negatively affected by the HFD, generating a significant reduction (*p* < 0.0001) in the activity of Δ5D and Δ-6D in the dams fed with HFD compared to the CD group (Figure 5D,E).

### 3.7. Hepatic mRNA Changes of PPAR-α and SREBP 1-c, and the Enzyme Regulated by These Transcription Factors from Offspring at 7 and 21 Days Fed with CD or HFD

**Figure 6:**
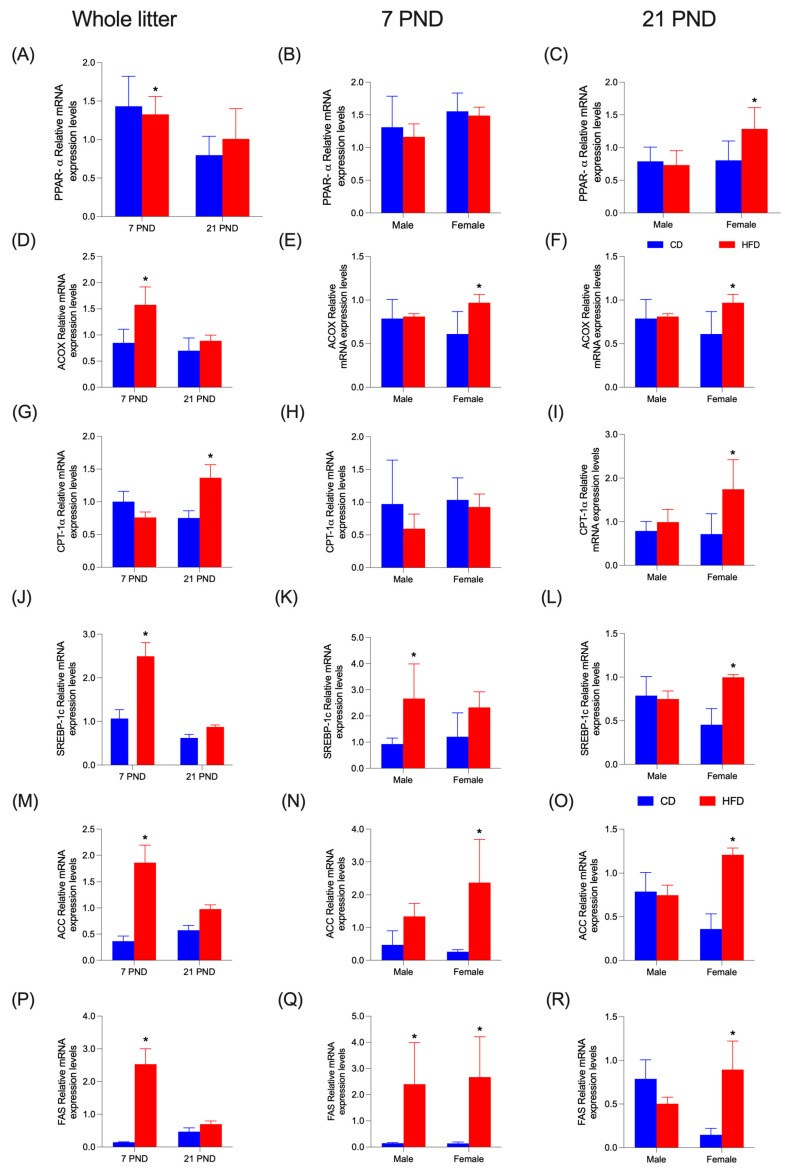
Relative mRNA expression in offspring liver at 7 and 21 days. (**A**–**C**) Expression of PPAR-α: Peroxisome proliferator-activated receptor alpha. (**D**–**F**) Expression of ACOX: Acyl-CoA oxidase. (**G**–**I**) Expression of CPT1-α: carnitine palmitoyl transferase 1α. (**J**–**L**) Expression of SREBP-1c: Sterol response element binding protein. (**M**–**O**) Expression of ACC: Acetyl-CoA carboxylase. (**P**–**R**) Expression of FAS: FA synthase. Data are expressed as mean ± SD. Significant differences between groups are indicated with *. Differences were calculated by two-way ANOVA followed by Sidák (*p* < 0.05) between CD (n = 6) and HFD (n = 6).

**Figure 7:**
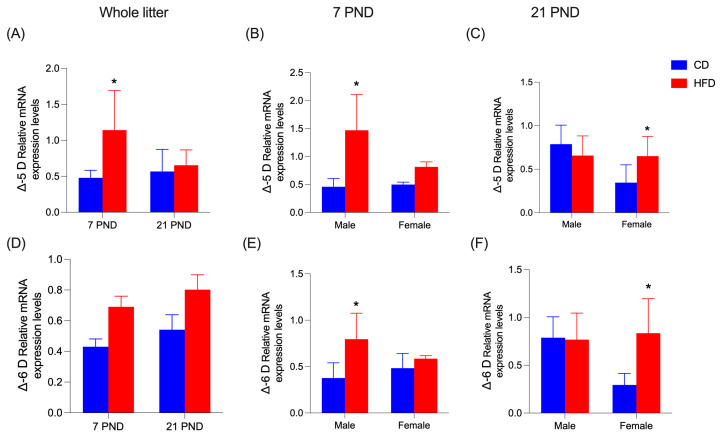
Relative mRNA expression in offspring liver at 7 and 21 days. (**A**–**C**) Expression of FADS-1, encodes Δ-5 Desaturase (Δ-5D). (**D**–**F**) Expression of FADS-2, encodes Δ-6 Desaturase (Δ-6D). Data are expressed as mean ± SD. Significant differences between groups are indicated with *. Differences were calculated by two-way ANOVA followed by Sidák (*p* < 0.05) between CD (n = 6) and HFD (n = 6).

Figure 6 displays hepatic mRNA levels of transcription factors PPAR-α and SREBP-1c, as well as enzymes ACOX, CPT-1α, ACC, and FAS. HFD diminishes (*p* = 0.0001) PPAR-α mRNA levels only in the whole litter at PND7 (Figure 6A); this situation reverts at PND21 in females (Figure 6C). No significant differences were found at PND7 (Figure 6B). In enzyme ACOX, mRNA levels were also increased by HFD (*p* < 0.0001), significantly at PND7 for the whole litter and in females for both PND7 and PND21 (Figure 6D–F). Enzyme CPT-1α mRNA levels only showed an increase (*p* = 0.005) in the entire litter at PND21 (Figure 6G) and females at PND21 (Figure 6I). At 7PND, no differences were observed (Figure 6H). At PND7, in the whole litter, HFD increased mRNA levels of SREBP-1c (*p* < 0.0001) (Figure 6J), ACC (*p* < 0.0001) (Figure 6M), and FAS (*p* < 0.0001) (Figure 6P). At 21PND in females, the same effect was observed in SREBP-1c (Figure 6L), ACC (*p* < 0.0001) (Figure 6O), and FAS (*p* < 0.0001) (Figure 6R). When detailed by sex, PND7 showed only an increase in males for SREBP-1c (*p* = 0.0119) (Figure 6K) and females for ACC (*p* = 0.0005) (Figure 6N). In the FAS enzyme, values for both males and females were increased by the HFD (*p* < 0.0001) (Figure 6Q). Hepatic mRNA levels of Δ-6D and Δ-5D are shown in Figure 7. Both Δ-6D and Δ-5D increased with HFD in males at PND7 (Figure 7B (*p* = 0.0004); Figure 7E (*p* = 0.021)) and females at PND21 (*p* = 0.009); (Figure 7C–F). In the whole litter, a rise was observed only in Δ-5D at PND7 (*p* = 0.0002); (Figure 7A). This effect was not observed at the same time in Δ-6D (Figure 7D).

### 3.8. Dam Behavior

**Figure 8:**
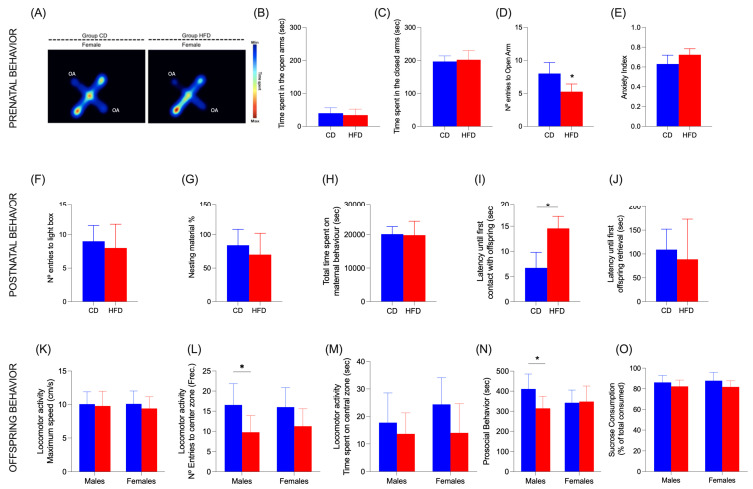
Dam behavioral results. (**A**) Combined heatmap generated by EthoVision^®^ Software XT 18 version (Noldus, Wageningen, The Netherlands) of Elevated Plus Maze (EPM) test in dams. OA: Open Arm. (**B**) Time spent in the open arms in EPM test. (**C**) Time spent in the closed arms in EPM test. (**D**) Number of entries to open arm in EPM test. (**E**) Anxiety index calculated according to Neto et al. [15]. (**F**) Number of entries to the light box in the Light Dark Box test. (**G**) Percentage of nesting material used by dams. (**H**) Total time spent on maternal behavior. (**I**) Latency until first contact with offspring in pup retrieval test. (**J**) Latency until first offspring retrieval in pup retrieval test. Values are expressed as media ± SD. Differences were calculated by Unpaired *t*-test with Welch’s correction (*p* < 0.05) between CD (n = 6) and HFD (n = 6). Offspring behavioral results: (**K**) Maximum speed in Open Field Test (OFT). (**L**) Number of entries to the center zone in Open Field Test (OFT). (**M**) Time spent in central zone in OFT. (**N**) Prosocial behavior in offspring: Time spent interacting with a novel rat. (**O**) Sucrose preference test. Data are expressed as mean ± SD. Significant differences between groups are indicated with *. Differences were calculated by two-way ANOVA followed by Sidák (*p* < 0.05) between male CD (n = 6), female CD (n = 6), male HFD (n = 6), and female HFD (n = 6).

Dam behavior was observed and measured through two tests. (i) EPM: This test was carried out during gestation. Figure 8A shows a representative image of a heat map. No significant differences between the groups were found in the time spent in open arms (Figure 8B) or closed arms (Figure 8C), although the frequency of entries to open arms shows a significant decrease (*p* = 0.008) in dams with HFD (Figure 8D). The anxiety index was also calculated according to Cohen et al. 2013 [28], but no differences were found (Figure 8E). (ii) LDB: Postpartum dams showed not significant differences in the number of entries to the light chamber (Figure 8F), neither in time spent in the light or dark chamber.

### 3.9. Maternal Behaviors

No significant differences were found in the use of nesting material provided by investigators (Figure 8G). The percentage of the time that dams spent performing maternal behaviors shows no differences between groups (Figure 8H). In the pup retrieval test, HFD increased the latency until first contact with offspring (*p* = 0.0004) (Figure 8I), but the delay was not observed in the time for the first retrieval (Figure 8J).

### 3.10. Offspring Behavior

(i) Open Field Test: The maximum speed in both males and females was not affected (Figure 8K) and the frequency of entries to the center zone decreased in males with HFD but not in females (*p* = 0.0276) (Figure 8L), and neither did the time spent on the central zone (Figure 8M). (ii) Social interaction: A significant difference was only found in males; HFD delayed the time the pups started prosocial behavior with the other pups (*p* = 0.0464) (Figure 8N). (iii) Sucrose preference test*:* The percentage of preference for sucrose solution about the water had no differences between diets (Figure 8O).

## 4. Discussion

During the perinatal period, the regular and sufficient flow of energy and nutrients is relevant to metabolic fetal programming, which can significantly impact neurological development in the first stage of life [5,6,9]. In this regard, our results exhibited that the intake of HFD during the pre- and gestational phases and postpartum and breastfeeding time generated a deleterious effect on metabolic and hepatic parameters, leading to the liver steatotic status, with significant changes in the molecular pathway involved in the FA metabolism (Figure 4 and Figure 5). Under this condition, this alteration negatively impacted the regular levels of *n*-3 PUFAs, specifically the ratio of *n*6:*n*3 in the different tissues, particularly in the brain (Table 4, Table 5, Table 6 and Table 7), generating a possible adverse consequence in the neurodevelopment [5,6,9,11].

In the dams, it is possible to find two principal aspects that directly participate in the FA nutritional status in the female and her offspring, (i) diet, and the (ii) excess of adipose tissue (or obesity) [6,29]. With respect to the diet, it has been documented that the maternal food intake during pregnancy and breastfeeding period has an important role in the FA composition in erythrocytes and breastmilk [30], the effect being more notable if the mother is obsese [31]. Regarding the nutritional status, it has been reported that the obesity reduced the synthesis and availability of *n*-3 PUFAs [13,32,33], because this pathology is directly related to development with liver steatosis, a condition that reduces the activity of Δ-5 D and Δ6-D [29]. Furthermore, obesity diminishes the placental transport of *n*-3 DHA disturbance that generates a lower availability of this FA in the fetus [6].

The type of dietary fats consumed plays a significant role in metabolic health [5,7,10]. Several research works indicate that *n*-3 PUFA can mitigate the adverse effects of a Western diet, particularly in the context of liver steatosis, by influencing hepatic FA metabolism [10,34,35]. In contrast, high intake of saturated fats, such as palmitic acid, has been associated with negative metabolic outcomes, including insulin resistance and dyslipidemia [36]. Maternal HFD during gestation and lactation period also has a profound effects on metabolic parameters in offspring. Research indicates that HFD exposure leads to significant alterations in body composition, including increased adiposity and impaired glucose tolerance in young animals [37,38]. Specifically, offspring of HFD-fed dams exhibit hyperglycemia and altered insulin sensitivity, which can be attributed to epigenetic modifications affecting gluconeogenic pathways (Table 1 and Table 2) [39].

While dietary intake is a primary determinant in FA profile changes, additional factors must be considered. First, hepatic function plays a crucial role in fatty acid metabolism, including elongation, desaturation, and β-oxidation. The liver is responsible for converting essential fatty acids into long-chain polyunsaturated fatty acids (LC-PUFAs) such as docosahexaenoic acid (DHA). Impairments in liver function could affect the efficiency of these metabolic pathways and alter the systemic fatty acid profile [29]. Second, the central nervous system (CNS) is a significant regulator of lipid homeostasis, particularly through its impact on peripheral metabolism via neuroendocrine signaling. The brain preferentially uptakes DHA, and changes in neuronal demand or blood–brain barrier transport mechanisms could modify the availability of circulating fatty acids. Furthermore, stress and hormonal fluctuations (e.g., glucocorticoids) have been shown to affect lipid metabolism, which may also contribute to changes in fatty acid profiles [40].

The FA composition in various tissues, particularly erythrocytes, liver, and brain, is crucial for metabolism and brain function [8,9]. Erythrocytes serve as a valuable indicator of FA status in the body, reflecting dietary intake and metabolic changes [30,35,41,42]. For instance, alterations in the FA composition of erythrocyte membranes have been linked to maternal dietary habits during pregnancy, which can affect fetal brain development [42]. Research indicates that higher levels of DHA in erythrocytes correlate with improved cognitive functions and brain health in later life, suggesting that maternal nutrition can have long-lasting effects on offspring brain function (Table 3) [42,43].

In the liver, the metabolism of FA is decisive for energy homeostasis and lipid storage. Overfeeding with different types of fats has been shown to cause distinct metabolic responses, leading to variations in liver fat accumulation and insulin sensitivity. For example, *n*-3 PUFAs can downregulate genes associated with fat storage, thereby influencing overall lipid metabolism and energy expenditure [10,40,44]. The brain’s FA composition is also critical for maintaining cognitive function and overall neurological health. Studies have demonstrated that a higher ratio of *n*-3:*n*-6 PUFA in the brain is associated with better cognitive performance and a lower risk of neurodegenerative diseases [43,44,45]. Conversely, an elevated *n*-6:*n*-3 ratio can promote inflammation and cognitive decline [46,47]. The FA composition in the brain is influenced by dietary intake, as well as the FA profiles of erythrocytes and liver, highlighting the interconnectedness of these tissues in regulating brain function and metabolism [43,48].

A HFD has been shown to significantly increase *de novo* lipogenesis (DNL) in the liver, which is a key factor in the development of fatty liver disease. Elevated levels of lipogenesis are often associated with insulin resistance and metabolic syndrome, both of which are exacerbated by HFD intake [49]. One of the primary mechanisms by which HFD induces lipogenesis is through the activation of specific transcription factors such as SREBP-1c [50]. This transcription factor plays a pivotal role in regulating the expression of genes involved in fatty acid synthesis [44]. Studies have demonstrated that HFD leads to the upregulation of SREBP-1c, which in turn enhances the expression of lipogenic enzymes such as fatty acid synthase (FAS) and acetyl-CoA carboxylase (ACC) [51]. This cascade results in increased fatty acid synthesis and subsequent accumulation of triglycerides in the liver, contributing to steatosis [52]. Moreover, the interplay between insulin signaling and lipogenesis is crucial in the context of HFD [50,51].

The impact of a HFD on PUFA metabolism is largely mediated by the regulation of key enzymes involved in their biosynthesis and conversion. Notably, the activities of Δ5- and Δ6-desaturases (FADS1 and FADS2) as well as elongases (ELOVL5 and ELOVL2) can be modulated by dietary lipid composition [13]. An excessive intake of saturated fatty acids and ω-6 PUFAs may competitively inhibit the enzymatic conversion of ALA to DHA, leading to a reduction in DHA availability [35]. Additionally, HFD-induced oxidative stress and endoplasmic reticulum homeostasis disruption can alter the expression of key transcription factors, such as SREBPs and PPARs, which play central roles in lipid metabolism regulation [29]. These alterations may impair the endogenous synthesis of long-chain PUFAs, thereby influencing their tissue distribution, particularly in the brain and liver [4,7,9,11]. Such metabolic shifts could have significant physiological implications, including consequences as neurodevelopmental issues, low-grade inflammation, and mood disorders [5,8,39].

Maternal HFD during pregnancy and lactation significantly influences offspring behavior and metabolic health, particularly regarding liver steatosis and fatty acid composition (Figure 4). Studies indicate that HFD exposure leads to increased liver triglycerides and cholesterol levels in offspring, promoting pro-lipogenic status [53,54]. Furthermore, maternal obesity alters the fatty acid profile in breastmilk, resulting in reduced levels of essential PUFAs in offspring (Table 5, Table 6 and Table 7), which are crucial for metabolic health [55,56]. It has been reported that offspring from HFD-fed dams exhibit altered appetitive responses, potentially due to changes in reward-processing pathways [57]. This altered behavior can predispose them to obesity and metabolic dysfunction later in life [58]. The interplay between maternal diet and offspring health underscores the importance of nutritional interventions during critical developmental windows to mitigate adverse outcomes associated with HFD exposure [59].

The maternal HFD not only affects metabolic health in offspring but also significantly influences maternal, anxiety-related, and offspring behaviors (Figure 8). While HFD intake did not affect some parameters of anxiety measures in pregnant dams, HFD did significantly decrease entries into the open arm in the EPM (Figure 8D), suggesting that the HFD diet had an anxiogenic effect for dams in the course of gestation that was corrected during lactation. This may be considered a minor issue; however, gestation is a sensitive period in the neurodevelopment of the offspring [60]. The amygdaloid complex in the brain area regulates anxiety as well as the activity of the hypothalamic–pituitary–adrenal (HPA) axis, which is associated with the release of stress hormones [61]. Corticosterone is the main stress hormone in rats, and through its receptors, glucocorticoid and mineralocorticoid, it plays a fundamental role in the development of brain structures that modulate anxiety, where mineralocorticoid receptors have a neuroprotective effect [62]. Thus, it is possible that the anxiogenic effect of the HFD during gestation could be translated into an increase in corticosterone levels in the brain. In this scenario during neurodevelopment, the male brain of the offspring is more vulnerable to the effects of corticosterone since it expresses low levels of MRs in the brain compared to the female brain [62]. This may in part explain why male pups showed an increase in anxiety-like behaviors compared to female pups (Figure 8L). There is evidence to suggest that HFD during gestation and lactation can lead to an increase in anxiety-like behaviors in offspring, particularly through alterations in the serotonergic system [58,59]. For instance, maternal obesity has been associated with dysregulation of the HPA axis, which is crucial for stress response and anxiety regulation [60,61]. Moreover, specific FA profiles in maternal diets, such as an imbalance between *n*-6 and *n*-3 PUFA, have been linked to heightened anxiety in offspring [62,63]. This relationship is further supported by findings that maternal exposure to HFD can lead to neuroinflammation, which is implicated in anxiety disorders [59]. Additionally, maternal androgen excess has been shown to induce sexually dimorphic anxiety-like behavior in offspring, highlighting the complex interplay between maternal diet, hormonal environment, and offspring behavior [24,63]. In this context, the anxiety induced by HFD during pregnancy may explain the impairment in maternal behavior of dams (Figure 8I). Moreover, the neglected maternal behavior is a strong stressor in early life that affects social behavior in rats [63], which may explain the decrease in prosocial behavior of male pups in our experiments (Figure 8N). In summary, maternal HFD is intricately connected to offspring anxiety through metabolic, neurochemical, and hormonal pathways, emphasizing the need for nutritional interventions during critical developmental periods.

## 5. Conclusions

The findings from this study highlight the significant impact of maternal HFD on both maternal and offspring health during a critical developmental period. HFD not only alters FA composition in liver, adipose tissues, and erythrocytes, but also leads to metabolic disturbances such as higher insulinemia, triglycerides, and liver enzyme levels in offspring. Additionally, the study reveals behavioral changes. An increase in anxiety-like behaviors in dams and reduction in social interactions in male offspring were observed, which may be linked to diet. Furthermore, the observed gender differences in response to HFD suggest that male and female offspring may be differentially affected by maternal dietary choices, highlighting the need for tailored nutritional interventions. The increased *n*-6:*n*-3 ratio in HFD groups points to potential imbalances that could predispose offspring to obesity and metabolic disorders later in life. These results emphasize the complex relationship between maternal nutrition and the long-term health outcomes of offspring. The Sprague–Dawley rat model provides valuable mechanistic insights but has limitations in translating findings to humans due to differences in lipid metabolism, brain development, and behavior. Controlled experimental conditions do not fully replicate human pregnancies and maternal nutrition. Thus, caution is needed when applying these results to human health. Overall, this study contributes to a growing body of evidence that stresses the importance of maternal diet during pregnancy and lactation, advocating for public health strategies aimed at improving maternal nutrition to promote better health outcomes for future generations.

## Figures and Tables

**Table 1:** Maternal metabolic and liver parameters.

Parameters	CD (a)	HFD (b)	*p* Value
Glycemia (mg/dL)	171 ± 24	180 ± 74	0.8053
Insulin (μU/mL)	7.7 ± 0.7	8.8 ± 0.4 ^b^	0.0376
HOMA-IR	3.2 ± 0.6	4.4 ± 1.6	0.2101
TG (mg/dL)	69 ± 21	105 ± 55	0.3606
T-Cho (mg/dL)	90 ± 14	103 ± 34	0.4545
HDL-c (mg/dL)	28 ± 7.3	29 ± 14	0.8563
GGT (IU/L)	10 ± 0.2	10 ± 0.5	0.3632
GPT (IU/L)	10 ± 0.1	11 ± 2.4	0.4610
GOT (IU/L)	57 ± 13	75 ± 37	0.3257

Values are expressed as median ± SD, n = 12. CD, control diet (n = 6); HFD, high-fat diet (n = 6). Unpaired *t*-test with Welch’s correction. Significant differences (*p* > 0.0001) between groups are indicated by the superscripted letter identifying each group (e.g., b); CD (a), HFD (b). TG, triacylglycerol; T-Cho, total cholesterol; HDL-c, high-density lipoprotein cholesterol; GGT, Gamma–glutamil transferase; GPT, Glutamat–Pyruvat-Transaminase; GOT, glutamic oxaloacetic transaminase.

**Table 2:** Metabolic and liver parameters from offspring obtained at 7 and 21 postnatal days.

	PND7			PND21		
Parameters	CD (a)	HFD (b)	*p* Value	CD (a)	HFD (b)	*p* Value
Glycemia (mg/dL)	182 ± 78	201 ± 38	0.7343	243 ± 98	270 ± 51	0.5998
Insulin (μU/mL)	6.2 ± 2.6	7.6 ± 1.6	0.2816	6.7 ± 1.6	7.6 ± 1.6	0.3205
HOMA-IR	2.1 ± 1.4	3.4 ± 0.7	0.1146	3.9 ± 1.1	5.3 ± 1.1	0.0616
TG (mg/dL)	179 ± 63	295 ± 13 ^b^	0.0058	80 ± 45	178 ± 83 ^b^	0.0493
T-Cho (mg/dL)	154 ± 19	145 ± 6.1	0.2916	116 ± 16	122 ± 11	0.5137
HDL-c (mg/dL)	58 ± 5.0	57 ± 4.0	0.6248	44 ± 8.3	48 ± 8.1	0.4645
GPT (IU/L)	10 ± 0.5	13 ± 3.0	0.0639	11 ± 1.2	17 ± 5.3 ^b^	0.0288
GGT (IU/L)	10 ± 0.5	11 ± 1.1 ^b^	0.0422	10 ± 0.2	10 ± 0.4	0.3632
GOT (IU/L)	60 ± 13	102 ± 45	0.0518	81 ± 19	82 ± 9.2	0.9306

Values are expressed as median ± SD, n = 12. CD, control diet (n = 6); HFD, high-fat diet (n = 6). CD (a), HFD (b). TG, triacylglycerol; T-Cho, total cholesterol; HDL-c, high-density lipoprotein cholesterol; GGT, Gamma–glutamil transferase; GPT, Glutamat–Pyruvat-Transaminase; GOT, glutamic oxaloacetic transaminase. Unpaired *t*-test with Welch’s correction. Significant differences (*p* < 0.05) between groups are indicated by the superscripted letter identifying each group (e.g., b).

**Table 3:** Determination of fatty acid composition in liver, adipose tissue, and erythrocytes from dams.

Fatty Acids (%mmol)	Liver			Adipose Tissue			Erythrocytes		
	CD (n = 6)	HFD (n = 6)	ANOVA *p*-Value	CD (n = 6)	HFD (n = 6)	ANOVA *p*-Value	CD (n = 6)	HFD (n = 6)	ANOVA *p*-Value
SFA									
C14:0	1.5 ± 0.2	1.5 ± 0.4	0.958	2.7 ± 0.4	5.3 ± 0.5 ^a^	<0.001	2.6 ± 0.4 ^a^	1.8 ± 0.3	0.004
C16:0	31.9 ± 3.2 ^a^	23.7 ± 2.2	<0.001	29.3 ± 4.0 ^a^	14.8 ± 1.7	<0.001	29.8 ± 1.6 ^a^	26.8 ± 1.3	0.004
C18:0	7.1 ± 1.5	15.3 ± 6.4 ^a^	0.012	3.7 ± 0.4	4.2 ± 2.3	0.634	18.9 ± 1.9	22.1 ± 1.2 ^a^	0.005
MUFA									
C 16:1	4.2 ± 1.1 ^a^	0.8 ± 0.4	<0.001	3.5 ± 1.4 ^a^	1.0 ± 0.4	0.002	0.7 ± 0.2 ^a^	0.5 ± 0.1	0.037
C18:1n9c	46.6 ± 3.5 ^a^	30.3 ± 7.4	<0.001	51.1 ± 2.6	47.2 ± 3.4	0.052	12.6 ± 0.9 ^a^	10.3 ± 1.1	0.002
PUFA									
C18:2*n*-6c (LA)	4.9 ± 2.0	20.0 ± 3.7 ^a^	<0.001	8.4 ± 1.6	24.5 ± 2.0 ^a^	<0.001	6.0 ± 0.8	10.7 ± 0.4 ^a^	<0.001
C18:3*n*-3 (ALA)	0.4 ± 0.1	0.8 ±0.1 ^a^	<0.001	0.1 ± 0.1	0.6 ± 0.1 ^a^	<0.001	0.6 ± 0.1 ^a^	0.4 ± 0.1	0.001
C20:4*n*-6 (AA)	0.3 ± 0.2	1.0 ± 0.4 ^a^	0.007	0.4 ± 0.2	1.0 ± 0.2 ^a^	<0.001	22.2 ± 1.0	21.1 ± 1.4	0.169
C22:5*n*-6 (DPA)	0.2 ± 0.1	0.2 ± 0.1	0.522	0.1 ± 0.1	0.03 ± 0.02	0.161	0.6 ± 0.2 ^a^	0.3 ± 0.1	0.025
C22:5*n*-3 (DPA)	0.2 ± 0.1	0.2 ± 0.1	0.575	0.1 ± 0.1	0.2 ± 0.2	0.335	1.6 ± 1.1	1.9 ± 1.0	0.622
C20:5*n*-3 (EPA)	0.2 ± 0.1	0.7 ± 0.2 ^a^	<0.001	0.1 ± 0.03	0.2 ± 0.04 ^a^	<0.001	1.3 ± 0.2	1.5 ± 0.2	0.209
C22:6*n*-3 (DHA)	2.6 ± 0.2	5.4 ± 0.6 ^a^	<0.001	0.2 ± 0.03	0.2 ± 0.04 ^a^	0.025	3.1 ± 0.5	4.2 ± 0.7 ^a^	0.008
ΣSFA	40.4 ± 2.3	40.5 ± 7.4	0.981	35.9 ± 4.0	24.4 ± 3.9 ^a^	<0.001	51.3 ± 0.9	50.7 ± 2.3	0.537
ΣMUFA	50.7 ± 3.1 ^a^	31.1 ± 7.4	<0.001	54.7 ± 3.0 ^a^	48.6 ± 2.9	0.005	13.3 ± 1.0 ^a^	10.8 ± 1.2	0.002
ΣPUFA	8.8 ± 2.2	28.4 ± 3.2 ^a^	<0.001	9.4 ± 1.4	27.0 ± 1.4 ^a^	<0.001	35.4 ± 1.0	40.2 ± 1.7 ^a^	<0.001
Σ*n*-6 PUFA	5.4 ± 2.0	21.3 ± 3.4 ^a^	<0.001	9.0 ± 1.4	25.8 ± 1.7 ^a^	<0.001	28.8 ± 1.0	32.2 ± 1.6 ^a^	0.001
Σ*n*-3 PUFA	3.4 ± 0.2	7.1 ± 0.7 ^a^	<0.001	0.5 ± 0.2	1.2 ± 0.4 ^a^	0.001	6.6 ± 0.8	8.0 ± 1.3 ^a^	0.044
Ratio *n*-6:*n*-3	1.6 ± 0.5	3.0 ± 0.7 ^a^	0.002	24.6 ± 15.1	23.2 ± 8.1	0.848	4.4 ± 0.6	4.1 ± 0.8	0.515

Values are expressed as % mmol of fatty acid methyl esters (FAMEs). Data are presented as mean ± SD. CD: control diet (n = 6); HFD: high-fat diet (n = 6). SFA: Saturated fatty acids correspond to 14:0, 16:0 and 18:0; MUFA: Monounsaturated fatty acids correspond to 16:1 and 18:1, *n*-9.; PUFA: Polyunsaturated fatty acids correspond to 18:2, *n*-6, 18:3, *n*-3, 20:4, *n*-6, 20:5, *n*-3, 22:5, *n*-3, and 22:6, *n*-3; *n*-6; *n*-6:*n*-3 ratio: 20:4, *n*-6: (20:5 *n*-3, 22:5 *n*-3 and 22:6 *n*-3). Differences were calculated by Sidák post-test *p* < 0.05 between CD and HFD. Values sharing the same letter in each row are not statistically significant.

**Table 4:** Fatty acid composition in liver tissue in male and female offspring at PND7 and PND21.

Fatty Acids (%mmol)	Males						Females					
	PND7			PND21			PND7			PND21		
	CD (n = 6)	HFD (n = 6)	ANOVA *p*-Value	CD (n = 6)	HFD (n = 6)	ANOVA *p*-Value	CD (n = 6)	HFD (n = 6)	ANOVA *p*-Value	CD (n = 6)	HFD (n = 6)	ANOVA *p*-Value
SFA												
C14:0	2.6 ± 0.8	3.0 ± 0.3	0.295	3.3 ± 0.4	2.6 ± 0.2	0.089	4.4 ± 0.3 ^a^	1.7 ± 0.1	<0.001	3.6 ± 0.5 ^a^	2.5 ± 0.3	<0.001
C16:0	33.9 ± 2.3 ^a^	21.2 ± 1.5	<0.001	25.9 ± 2.8 ^a^	18.3 ± 1.3	<0.001	32.1 ± 2.4 ^a^	24.2 ± 1.9	<0.001	28.3 ± 1.9 ^a^	20.0 ± 1.7	<0.001
C18:0	10.3 ± 1.1	11.8 ± 1.6	0.181	14.05 ± 1.07	17.3 ± 1.7 ^a^	0.001	9.9 ± 0.8	14.7 ± 1.7 ^a^	<0.001	12.1 ± 1.5	14.8 ± 1.0 ^a^	0.004
MUFA												
C16:1	0.9 ± 0.3 ^a^	0.4 ± 0.04	<0.012	1.4 ± 0.4 ^a^	0.3 ± 0.03	<0.001	1.4 ± 0.1 ^a^	0.4 ± 0.1	<0.001	1.8 ± 0.3 ^a^	0.5 ± 0.1	<0.001
C18:1n9c	28.3 ± 3.0	27.4 ± 2.2	0.741	27.1 ± 2.6 ^a^	22.4 ± 1.6	0.006	30.6 ± 3.9	29.2 ± 3.8	0.704	29.1 ± 2.6	25.4 ± 2.2	0.123
PUFA												
C18:2*n*-6c (LA)	13.2 ± 2.4	22.5 ± 1.6 ^a^	<0.001	12.4 ± 1.3	27.7 ± 2.2 ^a^	<0.001	13.7 ± 2.9	17.6 ± 1.6 ^a^	0.005	13.5 ± 1.1	26.6 ± 1.9 ^a^	<0.001
C18:3*n*-3 (ALA)	1.1 ± 0.1	2.3 ± 0.2 ^a^	<0.001	1.9 ± 0.1 ^a^	3.3 ± 0.3	<0.001	1.2 ± 0.3	3.6 ± 0.3 ^a^	<0.001	1.4 ± 0.4	3.2 ± 0.3 ^a^	<0.001
C20:4*n*-6 (AA)	1.0 ± 0.1	1.3 ± 0.1 ^a^	0.001	0.7 ± 0.1	1.0 ± 0.07 ^a^	<0.001	1.1 ± 0.2	0.9 ± 0.2	0.145	0.9 ± 0.1	0.8 ± 0.08	0.486
C22:5*n*-6 (DPA)	1.2 ± 0.4 ^a^	0.6 ± 0.1	0.014	1.0 ± 0.5 ^a^	0.3 ± 0.05	0.004	0.8 ± 0.1 ^a^	0.5 ± 0.1	<0.001	0.3 ± 0.1	0.2 ± 0.02	0.113
C22:5*n*-3 (DPA)	1.4 ± 0.3	1.2 ± 0.2	0.084	1.0 ± 0.1	1.0 ± 0.07	0.825	1.0 ± 0.1 ^a^	0.6 ± 0.07	<0.001	1.2 ± 0.2 ^a^	0.7 ± 0.07	<0.001
C20:5*n*-3 (EPA)	1.1 ± 0.1 ^a^	0.5 ± 0.1	<0.001	0.7 ± 0.1 ^a^	0.3 ± 0.06	<0.001	1.2 ± 0.1 ^a^	0.4 ± 0.05	<0.001	0.5 ± 0.1	0.6 ± 0.2	0.320
C22:6*n*-3 (DHA)	4.2 ± 0.8 ^a^	9.0 ± 1.4	<0.001	9.9 ± 1.4 ^a^	6.7 ± 0.5	<0.001	3.8 ± 0.8	7.4 ± 1.7 ^a^	<0.001	6.9 ± 0.8	5.9 ± 0.4	0.253
ΣSFA	46.9 ± 3.1 ^a^	36.1 ± 3.0	<0.001	43.3 ± 4.2 ^a^	38.3 ± 3.0	0.038	46.4 ± 3.2 ^a^	40.7 ± 3.7	0.013	44.1 ± 3.2 ^a^	37.4 ± 2.8	0.003
ΣMUFA	29.3 ± 2.8 ^a^	27.8 ± 2.2	0.502	28.6 ± 2.6 ^a^	22.8 ± 1.6	<0.001	32.0 ± 4.0	29.6 ± 3.7	0.396	30.9 ± 2.9 ^a^	26.0 ± 2.3	0.037
ΣPUFA	23.7 ± 2.2	37.6 ± 2.7 ^a^	<0.001	28.0 ± 3.2	40.5 ± 2.9 ^a^	<0.001	23.1 ± 2.0	31.2 ± 3.9 ^a^	<0.001	24.8 ± 1.6	38.2 ± 2.7 ^a^	<0.001
Σ*n*-6 PUFA	15.6 ± 2.5	24.5 ± 1.8 ^a^	<0.001	14.2 ± 1.6	29.0 ± 2.2 ^a^	<0.001	15.7 ± 2.8	19.1 ± 1.9 ^a^	0.021	14.8 ± 1.3	27.6 ± 2.0 ^a^	<0.001
Σ*n*-3 PUFA	8.1 ± 1.2	13.1 ± 1.5 ^a^	<0.001	13.7 ± 1.7 ^a^	11.4 ± 0.9	0.020	7.3 ± 1.4	12.1 ± 2.0 ^a^	<0.001	10.0 ± 1.0	10.5 ± 1.0	0.816
Ratio *n*-6:*n*-3	1.9 ± 0.6	1.8 ± 0.2	0.854	1.0 ± 0.08	2.5 ± 0.1 ^a^	<0.001	2.2 ± 0.8 ^a^	1.6 ± 0.1	0.035	1.4 ± 0.2	2.6 ± 0.2 ^a^	<0.001

Values are expressed as % mmol of fatty acid methyl esters (FAMEs). Data are presented as mean ± SD. CD: control diet (n = 6); HFD: high-fat diet (n = 6). Identification of saturated and unsaturated fatty acids and their relationships are shown in Table 3. Differences were calculated by two-way ANOVA analysis followed by Sidák post-test *p* < 0.05 between CD and HFD. Values sharing the same letter in each row are not statistically significant.

**Table 5:** Determination of fatty acids by gas chromatography in adipose tissue in male and female offspring at PND7 and PND21.

Fatty Acids (%mmol)	Males						Females					
	PND7			PND21			PND7			PND21		
	CD (n = 6)	HFD (n = 6)	ANOVA *p*-Value	CD (n = 6)	HFD (n = 6)	ANOVA *p*-Value	CD (n = 6)	HFD (n = 6)	ANOVA *p*-Value	CD (n = 6)	HFD (n = 6)	ANOVA *p*-Value
SFA												
C14:0	13.5 ± 2.1 ^a^	5.5 ± 0.4	<0.001	10.7 ± 1.0 ^a^	7.0 ± 0.8	<0.001	13.0 ± 0.9 ^a^	7.4 ± 0.7	<0.001	9.7 ± 0.9 ^a^	2.0 ± 0.1	<0.001
C15:0	0.2 ± 0.04	1.1 ± 0.5 ^a^	0.010	1.4 ± 0.9	0.9 ± 0.3	0.322	0.2 ± 0.1	1.0 ± 0.3	0.072	10.3 ± 1.0 ^a^	0.8 ± 0.6	<0.001
C16:0	25.2 ± 6.6 ^a^	14.2 ± 1.1	0.001	26.8 ± 6.1 ^a^	18.6 ± 1.5	0.011	28.8 ± 4.6 ^a^	16.2 ± 1.2	<0.001	25.1 ± 2.2 ^a^	18.5 ± 3.8	<0.001
C16:1	4.3 ± 0.6 ^a^	0.5 ± 0.14	<0.001	5.3 ± 1.1 ª	1.8 ± 0.4	<0.001	3.9 ± 0.3 ^a^	0.4 ± 0.04	<0.001	3.7 ± 0.3 ^a^	0.4 ± 0.1	0.017
C18:0	4.1 ± 0.3	5.6 ± 0.4	0.070	4.7 ± 1.0	19.9 ± 1.9 ^a^	<0.001	5.2 ± 0.7	9.4 ± 2.4 ^a^	0.017	3.5 ± 0.3	10.3 ± 4.3 ^a^	<0.001
MUFA												
C16:1	4.3 ± 0.6 ^a^	0.5 ± 0.14	<0.001	5.3 ± 1.1 ª	1.8 ± 0.4	<0.001	3.9 ± 0.3 ^a^	0.4 ± 0.04	<0.001	3.7 ± 0.3 ^a^	0.4 ± 0.1	0.017
C18:1n9c	42.4 ± 5.9	42.8 ± 3.5	0.986	38.5 ± 3.3 ^a^	26.3 ± 3.4	<0.001	39.2 ± 2.8	41.0 ± 3.4	0.457	37.9 ± 2.8	43.5 ± 6.3	0.057
PUFA												
C18:2*n*-6c (LA)	6.3 ± 0.5	23.6 ± 1.9 ^a^	<0.001	10.2 ± 2.4	25.7 ± 1.9 ^a^	<0.001	6.9 ± 0.5	19.3 ± 3.2 a	<0.001	7.4 ± 0.9	24.6 ± 5.6 ^a^	<0.001
C18:3*n*-3 (ALA)	0.6 ± 0.04 ^a^	0.5 ± 0.05	<0.001	0.1 ± 0.03	0.2 ± 0.01	0.055	0.5 ± 0.04 ^a^	0.3 ± 0.1	0.006	0.1 ± 0.01	0.2 ± 0.1	0.098
C20:1n9	0.4 ± 0.2	0.4 ± 0.3	0.986	0.2 ± 0.1	0.02 ± 0.01	0.191	1.0 ± 0.4 ^a^	0.1 ± 0.03	<0.001	0.2 ± 0.1	0.2 ± 0.1	0.931
C20:2	0.4 ± 0.1	0.6 ± 0.2 ^a^	0.029	0.3 ± 0.07	0.3 ± 0.2	0.962	1.0 ± 0.4 ^a^	0.1 ± 0.03	<0.001	0.2 ± 0.04	0.1 ± 0.1	0.865
C20:4*n*-6 (AA)	1.5 ± 0.2	1.4 ± 0.3	0.838	0.8 ± 0.2	0.6 ± 0.05	0.438	1.7 ± 0.1	1.5 ± 0.1	0.172	0.6 ± 0.1	0.5 ± 0.2	0.246
C22:5*n*-6 (DPA *n*-6)	0.1 ± 0.04	0.3 ± 0.1 ^a^	<0.001	0.05 ± 0.03	0.04 ± 0.01	0.980	0.1 ± 0.1 ^a^	0.05 ± 0.01	<0.001	0.1 ± 0.01	0.1 ± 0.01	0.977
C22:5*n*-3 (DPA *n*-3)	0.2 ± 0.1	0.4 ± 0.2 ^a^	0.017	0.1 ± 0.06	0.1 ± 0.004	0.647	0.2 ± 0.1 ^a^	0.1 ± 0.1	<0.001	0.1 ± 0.03	0.1 ± 0.01	0.265
C20:5*n*-3 (EPA)	0.3 ± 0.1	0.7 ± 0.1 ^a^	<0.001	0.3 ± 0.1 ^a^	0.1 ± 0.01	0.003	0.3 ± 0.1	0.9 ± 0.1 ^a^	<0.001	0.3 ± 0.1	0.2 ± 0.05	0.012
C22:6*n*-3 (DHA)	0.5 ± 0.2	0.6 ± 0.1	0.059	0.6 ± 0.2 ^a^	0.2 ± 0.01	<0.001	0.6 ± 0.1	0.5 ± 0.1	0.777	0.7 ± 0.2 ^a^	0.2 ± 0.03	<0.001
ΣSFA	43.0 ± 5.7 ^a^	26.4 ± 2.2	<0.001	43.6 ± 4.2	46.4 ± 3.3	0.425	47.2 ± 4.8 ^a^	34.0 ± 3.5	0.001	48.6 ± 3.9 ^a^	31.6 ± 8.7	<0.001
ΣMUFA	47.1 ± 6.1 ^a^	43.7 ± 3.8	0.330	44.0 ± 3.3 ^a^	28.1 ± 3.1	<0.001	43.1 ± 3.2	41.5 ± 3.5	0.764	41.8 ± 3.2	44.1 ± 6.3	0.590
ΣPUFA	9.9 ± 0.7	28.2 ± 2.4 ^a^	<0.001	12.4 ± 2.6	27.1 ± 1.9 ^a^	<0.001	11.4 ± 0.8	22.8 ± 3.6 ^a^	<0.001	9.6 ± 0.9	26.0 ± 5.5 ^a^	<0.001
Σ*n*-6 PUFA	8.3 ± 0.6	26.0 ± 2.1 ^a^	<0.001	11.3 ± 2.7	26.7 ± 1.9 ^a^	<0.001	9.8 ± 0.8	20.9 ± 3.4 ^a^	<0.001	8.3 ± 1.0	25.3 ± 5.5 ^a^	<0.001
Σ*n*-3 PUFA	1.6 ± 0.4	2.2 ± 0.4 ^a^	0.004	1.1 ± 0.3 ^a^	0.5 ± 0.03	0.006	1.6 ± 0.3	1.9 ± 0.2	0.080	1.3 ± 0.2 ^a^	0.7 ± 0.1	<0.001
Ratio *n*-6:*n*-3	5.5 ± 1.6	11.8 ± 1.7 ^a^	<0.001	11.1 ± 4.1	58.1 ± 0.8 ^a^	<0.001	6.2 ± 1.5	11.1 ± 0.8	0.224	6.6 ± 1.6	36.5 ± 10.0 ^a^	<0.001

Values are expressed as % mmol of fatty acid methyl esters (FAMEs). Data are presented as mean ± SD. CD: control diet (n = 6); HFD: high-fat diet (n = 6). Identification of saturated and unsaturated fatty acids and their relationships are shown in Table 3. CD: control diet; HFD: high-fat diet. Differences were calculated by two-way ANOVA analysis followed by Sidák post-test *p* < 0.05 between CD and HFD. Values sharing the same letter in each row are not statistically significant.

**Table 6:** Determination of fatty acids by gas chromatography in erythrocytes in male and female offspring at PND7 and PND21.

Fatty Acids (%mmol)	Males						Females					
	PND7			PND21			PND7			PND21		
	CD (n = 6)	HFD (n = 6)	ANOVA *p*-Value	CD (n = 6)	HFD (n = 6)	ANOVA *p*-Value	CD (n = 6)	HFD (n = 6)	ANOVA *p*-Value	CD (n = 6)	HFD (n = 6)	ANOVA *p*-Value
SFA												
C14:0	3.3 ± 1.1	3.0 ± 0.8	0.811	4.2 ± 1.0 ^a^	1.8 ± 0.4	<0.001	2.9 ± 0.8	3.9 ± 1.3	0.100	2.4 ± 0.6	1.9 ± 0.7	0.576
C16:0	34.2 ± 4.6	30.3 ± 2.5	0.127	35.4 ± 4.0 ^a^	29.3 ± 2.1	0.011	37.7 ± 2.8	30.5 ^a^ ± 2.6	<0.001	35.2 ± 3.1 ^a^	28.4 ± 2.1	<0.001
C18:0	11.8 ± 1.6	14.9 ± 2.0 ^a^	0.006	12.6 ± 1.1	19.6 ± 1.5 ^a^	<0.001	11.9 ± 0.9	13.9 ± 1.3 ^a^	0.045	16.7 ± 1.2	20.3 ± 2.1 ^a^	<0.001
MUFA												
C16:1	1.5 ± 0.3 ^a^	0.6 ± 0.1	<0.001	1.5 ± 0.5 ^a^	0.3 ± 0.02	<0.001	1.0 ± 0.1	0.9 ± 0.2	0.334	1.1 ± 0.3 ^a^	0.5 ± 0.1	<0.001
C18:1n9c	25.6 ± 2.9 ^a^	19.4 ± 2.1	<0.001	22.6 ± 2.9 ^a^	14.5 ± 1.5	<0.001	20.7 ± 1.5	20.0 ± 1.7	0.660	18.0 ± 1.8 ^a^	15.5 ± 1.2	0.021
PUFA												
C18:2*n*-6c (LA)	9.9 ± 1.6	15.2 ± 2.0 ^a^	<0.001	10.8 ± 1.3	14.5 ± 1.0 ^a^	0.001	8.1 ± 1.8	16.4 ± 1.4 ^a^	<0.001	12.3 ± 1.8	13.2 ± 1.4	0.558
C18:3*n*-3 (ALA)	0.9 ± 0.2	1.2 ± 0.2 ^a^	0.001	1.3 ± 0.1	1.7 ± 0.2 ^a^	<0.001	0.7 ± 0.05	0.7 ± 0.1	0.796	0.9 ± 0.2	1.4 ± 0.3 ^a^	<0.001
C20:4*n*-6 (AA)	7.9 ± 0.8	12.7 ± 2.8 ^a^	<0.001	6.5 ± 1.8	15.0 ± 1.6 ^a^	<0.001	10.3 ± 0.9	11.1 ± 1.1	0.263	9.4 ± 0.6	12.5 ± 1.0 ^a^	<0.001
C22:5*n*-6 (DPA)	0.7 ± 0.4 ^a^	0.3 ± 0.04	0.009	0.6 ± 0.3 ^a^	0.2 ± 0.02	0.037	0.4 ± 0.1 ^a^	0.3 ± 0.1	0.021	0.5 ± 0.1	0.5 ± 0.04	0.687
C22:5*n*-3 (DPA)	1.1 ± 0.4	0.8 ± 0.1	0.128	0.9 ± 0.4	0.8 ± 0.1	0.843	0.9 ± 0.1 ^a^	0.6 ± 0.1	0.003	0.9 ± 0.1	0.9 ± 0.2	0.945
C20:5*n*-3 (EPA)	1.1 ± 0.4	1.3 ± 0.2	0.303	0.6 ± 0.1	1.5 ± 0.2 ^a^	<0.001	1.1 ± 0.1	1.1 ± 0.1	0.856	1.0 ± 0.1	1.5 ± 0.2 ^a^	<0.001
C22:6*n*-3 (DHA)	2.0 ± 0.2	2.0 ± 0.5	0.999	1.4 ± 0.6	2.3 ± 0.2 ^a^	0.002	2.7 ± 0.2 ^a^	2.2 ± 0.2	<0.001	1.7 ± 0.1	1.8 ± 0.2	0.350
ΣSFA	49.4 ± 4.9	48.3 ± 4.1	0.893	52.3 ± 5.1	50.7 ± 3.6	0.798	52.5 ± 4.0	48.3 ± 3.6	0.171	54.3 ± 4.7	50.6 ± 3.8	0.232
ΣMUFA	27.1 ± 2.8 ^a^	20.0 ± 2.2	<0.001	24.1 ± 3.4 ^a^	14.8 ± 1.5	<0.001	21.8 ± 1.6	20.9 ± 1.6	0.591	19.0 ± 2.0 ^a^	15.9 ± 1.2	0.007
ΣPUFA	23.5 ± 2.8	33.4 ± 2.9 ^a^	<0.001	22.0 ± 4.5	36.2 ± 3.0 ^a^	<0.001	24.1 ± 2.1	32.5 ± 2.9 ^a^	<0.001	26.6 ± 2.5	31.8 ± 3.0 ^a^	0.005
Σ*n*-6 PUFA	18.5 ± 1.9	28.1 ± 2.2 ^a^	<0.001	17.9 ± 3.3	29.8 ± 2.4 ^a^	<0.001	18.8 ± 1.8	27.8 ± 2.6 ^a^	<0.001	22.2 ± 2.1	26.2 ± 2.4 ^a^	0.010
Σ*n*-3 PUFA	5.0 ± 0.9	5.3 ± 0.9	0.840	4.1 ± 1.2	6.4 ± 0.7 ^a^	<0.001	5.3 ± 0.4 ^a^	4.7 ± 0.3	0.038	4.5 ± 0.4	5.6 ± 0.6 ^a^	<0.001
Ratio *n*-6:*n*-3	3.7 ± 0.4	5.4 ± 0.8 ^a^	<0.001	4.5 ± 0.6	4.7 ± 0.2	0.803	3.5 ± 0.2	5.9 ± 0.2 ^a^	<0.001	5.0 ± 0.1 ^a^	4.7 ± 0.1	0.034

Values are expressed as % mmol of fatty acid methyl esters (FAMEs). Data are presented as mean ± SD. CD: control diet (n = 6); HFD: high-fat diet (n = 6). Identification of saturated and unsaturated fatty acids and their relationships are shown in Table 3. Differences were calculated by two-way ANOVA analysis followed by Sidák post-test *p* < 0.05 between CD and HFD. Values sharing the same letter in each row are not statistically significant.

**Table 7:** Determination of fatty acids by gas chromatography in brain in males and female offspring at PND7 and PND21.

Fatty Acids (%mmol)	Males						Females					
	PND7			PND21			PND7			PND21		
	CD (n = 6)	HFD (n = 6)	ANOVA *p*-Value	CD (n = 6)	HFD (n = 6)	ANOVA *p*-Value	CD (n = 6)	HFD (n = 6)	ANOVA *p*-Value	CD (n = 6)	HFD (n = 6)	ANOVA *p*-Value
SFA												
C14:0	6.0 ± 1.3 ^a^	3.3 ± 0.3	<0.001	2.9 ± 0.7	3.1 ± 0.3	0.849	3.6 ± 0.3	3.6 ± 0.4	0.881	3.2 ± 0.3	2.9 ± 0.2	0.171
C16:0	34.3 ± 2.3	34.8 ± 2.2	0.910	29.6 ± 1.9	29.8 ± 2.1	0.987	35.9 ± 2.7	34.1 ± 2.4	0.360	28.9 ± 1.9	29.2 ± 1.9	0.979
C18:0	16.0 ± 1.1	17.2 ± 1.1	0.296	21.1 ± 1.5	21.9 ± 1.7	0.493	17.0 ± 1.3	16.2 ± 1.1	0.524	20.8 ± 1.4	20.9 ± 1.3	0.992
MUFA												
C16:1	2.9 ± 0.4 ^a^	2.2 ± 0.3	<0.001	1.4 ± 0.3 ^a^	0.7 ± 0.1	<0.001	2.5 ± 0.2	5.1 ± 2.6 ^a^	0.004	0.8 ± 0.1	1.1 ± 0.5	0.890
C18:1n9c	12.4 ± 0.9	12.5 ± 0.8	0.986	14.9 ± 1.0	16.4 ± 1.2 ^a^	0.032	13.1 ± 1.0	12.6 ± 0.8	0.728	16.8 ± 1.1	15.9 ± 1.2	0.278
PUFA												
C18:2*n*-6c (LA)	2.3 ± 0.3	4.5 ± 0.7 ^a^	<0.001	2.1 ± 0.2	1.9 ± 0.2	0.677	1.6 ± 0.1	4.7 ± 0.5 ^a^	<0.001	1.5 ± 0.2	1.8 ± 0.2	0.147
C18:3*n*-3 (ALA)	0.3 ± 0.02	0.4 ± 0.1 ^a^	0.002	0.4 ± 0.1	0.8 ± 0.1 ^a^	<0.001	0.3 ± 0.02	0.3 ± 0.1	0.471	0.6 ± 0.1	0.6 ± 0.04	0.916
C20:4*n*-6 (AA)	12.7 ± 1.0	12.5 ± 0.9	0.927	12.5 ± 0.9	12.1 ± 1.1	0.687	14.0 ± 1.0 ^a^	11.6 ± 1.2	<0.001	12.6 ± 0.8	12.6 ± 0.8	0.994
C22:5*n*-6 (DPA)	2.2 ± 0.2 ^a^	1.6 ± 0.2	<0.001	1.7 ± 0.2 ^a^	1.3 ± 0.2	0.002	2.4 ± 0.2 ^a^	1.2 ± 0.2	<0.001	1.6 ± 0.1 ^a^	1.2 ± 0.1	0.001
C22:5*n*-3 (DPA)	2.7 ± 0.3	2.8 ± 0.5	0.934	2.9 ± 0.2	2.9 ± 0.2	0.999	2.9 ± 0.2 ^a^	2.5 ± 0.2	0.010	3.0 ± 0.2	3.1 ± 0.3	0.389
C20:5*n*-3 (EPA)	0.3 ± 0.1	0.2 ± 0.02	0.129	0.2 ± 0.03 ^a^	0.2 ± 0.01	0.004	0.2 ± 0.02	0.2 ± 0.02	0.305	0.2 ± 0.01	0.2 ± 0.03	0.459
C22:6*n*-3 (DHA)	7.8 ± 0.6	8.0 ± 0.7	0.801	10.2 ± 0.7	10.6 ± 0.8	0.495	8.0 ± 0.6	7.7 ± 0.8	0.720	10.0 ± 0.7	10.5 ± 0.7	0.451
ΣSFA	56.4 ± 3.6	55.3 ± 3.5	0.853	53.6 ± 3.5	54.9 ± 4.0	0.801	56.5 ± 4.2	53.9 ± 3.6	0.421	52.9 ± 3.4	52.9 ± 3.4	>0.999
ΣMUFA	15.3 ± 1.1	14.7 ± 1.0	0.515	16.3 ± 1.1	17.1 ± 1.2	0.428	15.5 ± 1.2	17.8 ± 3.0	0.084	17.6 ± 1.2	17.0 ± 1.2	0.835
ΣPUFA	28.3 ± 1.8	30.0 ± 1.9	0.285	30.1 ± 2.0	29.7 ± 2.4	0.941	29.4 ± 2.2	28.3 ± 2.3	0.603	29.5 ± 1.9	30.1 ± 1.9	0.875
Σ*n*-6 PUFA	17.2 ± 1.1	18.6 ± 1.2	0.157	16.3 ± 1.1	15.3 ± 1.5	0.284	18.0 ± 1.3	17.6 ± 1.4	0.802	15.7 ± 1.0	15.6 ± 1.0	0.986
Σ*n*-3 PUFA	11.0 ± 0.7	11.5 ± 0.9	0.662	13.8 ± 1.0	14.5 ± 1.0	0.365	11.4 ± 0.8	10.7 ± 1.0	0.375	13.8 ± 0.9	14.4 ± 0.9	0.406
Ratio *n*-6:*n*-3	1.6 ± 0.01	1.6 ± 0.1	0.324	1.2 ± 0.01 ^a^	1.1 ± 0.1	0.007	1.6 ± 0.0	1.6 ± 0.1 ^a^	0.005	1.1 ± 0.02 ^a^	1.1 ± 0.01	0.015

Values are expressed as % mmol of fatty acid methyl esters (FAMEs). Data are presented as mean ± SD. CD: control diet (n = 6); HFD: high-fat diet (n = 6). Identification of saturated and unsaturated fatty acids and their relationships are shown in Table 3. Differences were calculated by two-way ANOVA analysis followed by Sidák post-test *p* < 0.05 between CD and HFD. Values sharing the same letter in each row are not statistically significant.

## Data Availability

Additional data are to be shared upon request from the corresponding author (R.V.).

## Supplementary Materials

The following supporting information can be downloaded at: https://www.mdpi.com/article/10.3390/nu17071180/s1. Table S1. Diets composition. information provided by Research diet, New Jersey, USA. Table S2. Gene specific TacMan probes used in the study. Table S3. Determination of fatty acids by gas chromatography in stomach content from offspring at PND7.

## Abbreviations

HFD	High-Fat Diet
CD	Control Diet
DOHaD	Developmental Origins of Health and Disease
FA	Fatty Acid
PUFA	Polyunsaturated Fatty Acid
DHA	Docosahexaenoic Acid
ARA	Arachidonic Acid
ALA	Alpha-linolenic Acid
LA	Linoleic Acid
Δ5-D	Δ-5 Desaturase
Δ6-D	Δ-6 Ddesaturase
EPM	Elevated Plus Maze
LDB	Light Dark Box
PND	Post-Natal Day
*OFT*	Open Field Test
SPT	Sucrose Preference Test
HOMA	Homeostasis Model Assessment Method
TG	Triglycerides
T-Cho	Total Cholesterol
HDL-c	HDL-Cholesterol
GGT	Gamma–Glutamil-Transferase
GPT	Glutamat–Pyruvat-*Transaminase*
*GOT*	Glutamic Oxaloacetic *Transaminase*
SDA	Stearidonic Acid
DGLA	Dihomo-Gamma-Linolenic Acid
PPAR-α	Peroxisome Proliferator-Activated Receptor Alpha
ACOX	Acyl-CoA Oxidase
CPT1-α	Carnitine Palmitoyl Transferase 1α
SREBP-1c	Sterol Response Element Binding Protein
ACC	Acetyl-CoA-Carboxylase
FAS	FA Synthase
GAPDH	Glyceraldehyde 3-Phosphate Dehydrogenase
SFA	Saturated Fatty Acids
MA	Miristic Acid
PA	Palmitic Acid
SA	Stearic Acid
MUFA	Monounsaturated Fatty Acid
POA	Palmitoleic Acid
OA	Oleic Acid
*n*-6 DPA	*n*-6 Docosapentaenoic
*n*-3 DPA	*n*-3 Docosapentaenoic
DNL	*De Novo* Lipogenesis
HPA	Hypothalamic–Pituitary–Adrenal Axis

## References

[1] Denizli M., Capitano M.L., Kua K.L. (2022). Maternal obesity and the impact of associated early-life inflammation on long-term health of offspring. Front. Cell. Infect. Microbiol..

[2] Consales A., Morniroli D., Vizzari G., Mosca F., Giannì M.L. (2022). Nutrition for Infant Feeding. Nutrients.

[3] Abdelrahman M.A., Osama H., Saeed H., Madney Y.M., Harb H.S., Abdelrahim M.E.A. (2022). Impact of *n*-3 polyunsaturated fatty acid intake in pregnancy on maternal health and birth outcomes: Systematic review and meta-analysis from randomized controlled trails. Arch. Gynecol. Obstet..

[4] Álvarez D., Muñoz Y., Ortiz M., Maliqueo M., Chouinard-Watkins R., Valenzuela R. (2020). Impact of Maternal Obesity on the Metabolism and Bioavailability of Polyunsaturated Fatty Acids during Pregnancy and Breastfeeding. Nutrients.

[5] Ortiz M., Sánchez F., Álvarez D., Flores C., Salas-Pérez F., Valenzuela R., Cantin C., Leiva A., Crisosto N., Maliqueo M. (2023). Association between maternal obesity, essential fatty acids and biomarkers of fetal liver function. Prostaglandins Leukot. Essent. Fatty Acids.

[6] Gázquez A., Prieto-Sánchez M.T., Blanco-Carnero J.E., Ruíz-Palacios M., Nieto A., van Harskamp D., Oosterink J., Schierbeek H., van Goudoever J., Demmelmair H. (2020). Altered materno-fetal transfer of 13C-polyunsaturated fatty acids in obese pregnant women. Clin. Nutr..

[7] Chianese R., Coccurello R., Viggiano A., Scafuro M., Fiore M., Coppola G., Operto F.F., Fasano S., Laye S., Pierantoni R. (2017). Impact of Dietary Fats on Brain Functions. Curr. Neuropharmacol..

[8] Larrieu T., Layé S. (2018). Food for Mood: Relevance of Nutritional Omega-3 Fatty Acids for Depression and Anxiety. Front. Physiol..

[9] Martinat M., Rossitto M., Di Miceli M., Layé S. (2021). Perinatal dietary polyunsaturated fatty acids in brain development, role in neurodevelopmental disorders. Nutrients.

[10] Videla L.A., Hernandez-Rodas M.C., Metherel A.H., Valenzuela R. (2022). Influence of the nutritional status and oxidative stress in the desaturation and elongation of *n*-3 and *n*-6 polyunsaturated fatty acids: Impact on non-alcoholic fatty liver disease. Prostaglandins Leukot. Essent. Fatty Acids.

[11] Devarshi P.P., Grant R.W., Ikonte C.J., Hazels Mitmesser S. (2019). Maternal Omega-3 Nutrition, Placental Transfer and Fetal Brain Development in Gestational Diabetes and Preeclampsia. Nutrients.

[12] Zehravi M., Maqbool M., Ara I. (2021). Correlation between obesity, gestational diabetes mellitus, and pregnancy outcomes: An overview. Int. J. Adolesc. Med. Health.

[13] Valenzuela R., Metherel A.H., Cisbani G., Smith M.E., Chouinard-Watkins R., Klievik B.J., Videla L.A., Bazinet R.P. (2024). Protein concentrations and activities of fatty acid desaturase and elongase enzymes in liver, brain, testicle, and kidney from mice: Substrate dependency. BioFactors.

[14] Tung K.T.S., Wong R.S., Mak R.T.W. (2023). Maternal *n*-3 PUFA Intake During Pregnancy and Perinatal Mental Health Problems: A Systematic Review of Recent Evidence. Curr. Nutr. Rep..

[15] Neto J., Jantsch J., De Oliveira S., Braga M.F., Castro L.F.D.S., Diniz B.F., Moreira J.C.F., Giovenardi M., Porawski M., Guedes R.P. (2022). DHA/EPA supplementation decreases anxiety-like behaviour, but it does not ameliorate metabolic profile in obese male rats. Br. J. Nutr..

[16] Biała G.K.M. (2007). Amphetamine-induced anxiety-related behavior in animal models. Pharmacol. Rep..

[17] Matthews D.R., Hosker J.P., Rudenski A.S., Naylor B.A., Treacher D.F., Turner R.C. (1985). Homeostasis model assessment: Insulin resistance and cell function from fasting plasma glucose and insulin concentrations in man. Diabetologia.

[18] Brunt E.M., Janney C.G., Di Bisceglie A.M., Neuschwander-Tetri B.A., Bacon B.R. (1999). Nonalcoholic Steatohepatitis: A Proposal for Grading and Staging The Histological Lesions. Am. J. Gastroenterol..

[19] Bligh E.G., Dyer W.J. (1959). A rapid method of total lipid extraction and purification. Can. J. Biochem. Physiol..

[20] Morrison W.R., Smith L.M. (1964). Preparation of fatty acid methyl esters and dimethylacetals from lipids with boron fluoride–methanol. J. Lipid Res..

[21] Pfaffl M.W. (2001). A new mathematical model for relative quantification in real-time RT-PCR. Nucleic Acids Res..

[22] Moazzam S., Jarmasz J.S., Jin Y., Siddiqui T.J., Cattini P.A. (2021). Effects of high fat diet-induced obesity and pregnancy on prepartum and postpartum maternal mouse behavior. Psychoneuroendocrinology.

[23] Bosch O.J., Müsch W., Bredewold R., Slattery D.A., Neumann I.D. (2007). Prenatal stress increases HPA axis activity and impairs maternal care in lactating female offspring: Implications for postpartum mood disorder. Psychoneuroendocrinology.

[24] Aguggia J.P., Suárez M.M., Rivarola M.A. (2013). Early maternal separation: Neurobehavioral consequences in mother rats. Behav. Brain Res..

[25] Malkesman O., Weller A. (2009). Two different putative genetic animal models of childhood depression—A review. Prog. Neurobiol..

[26] Meaney M.J., Stewart J. (1981). Neonatal androgens influence the social play of prepubescent rats. Horm. Behav..

[27] Bravo-Tobar I.D., Fernández P., Sáez J.C., Dagnino-Subiabre A. (2021). Long-term effects of stress resilience: Hippocampal neuroinflammation and behavioral approach in male rats. J. Neurosci. Res..

[28] Cohen H., Matar M.A., Joseph Z. (2013). Animal Models of Post-Traumatic Stress Disorder. Curr. Protoc. Neurosci..

[29] Badmus O.O., Hillhouse S.A., Anderson C.D., Hinds T.D., Stec D.E. (2022). Molecular mechanisms of metabolic associated fatty liver disease (MAFLD): Functional analysis of lipid metabolism pathways. Clin. Sci..

[30] Barrera C., Valenzuela R., Chamorro R., Bascuñán K., Sandoval J., Sabag N., Valenzuela F., Valencia M.-P., Puigrredon C., Valenzuela A. (2018). The Impact of Maternal Diet during Pregnancy and Lactation on the Fatty Acid Composition of Erythrocytes and Breast Milk of Chilean Women. Nutrients.

[31] Chamorro R., Bascuñán K.A., Barrera C., Sandoval J., Puigrredon C., Valenzuela R. (2022). Reduced *n*-3 and *n*-6 PUFA (DHA and AA) Concentrations in Breast Milk and Erythrocytes Phospholipids during Pregnancy and Lactation in Women with Obesity. Int. J. Environ. Res. Public Health.

[32] Domenichiello A.F., Kitson A.P., Bazinet R.P. (2015). Is docosahexaenoic acid synthesis from α-linolenic acid sufficient to supply the adult brain?. Prog. Lipid Res..

[33] Domenichiello A.F., Kitson A.P., Chen C.T., Trépanier M.O., Stavro P.M., Bazinet R.P. (2016). The effect of linoleic acid on the whole body synthesis rates of polyunsaturated fatty acids from α-linolenic acid and linoleic acid in free-living rats. J. Nutr. Biochem..

[34] Garcia-Jaramillo M., Spooner M.H., Löhr C.V., Wong C.P., Zhang W., Jump D.B. (2019). Lipidomic and transcriptomic analysis of western diet-induced nonalcoholic steatohepatitis (NASH) in female Ldlr -/- mice. PLoS ONE.

[35] Wan F., Pan F., Mori T.A., O’Sullivan T.A., Beilin L.J., Oddy W.H. (2023). Relationship between dietary intake and erythrocyte PUFA in adolescents from a Western Australian cohort. Eur. J. Clin. Nutr..

[36] Murru E., Manca C., Carta G., Banni S. (2022). Impact of Dietary Palmitic Acid on Lipid Metabolism. Front. Nutr..

[37] Cavalcanti C.C.L., Manhães-de-Castro R., Chaves W.F., Cadena-Burbano E.V., Antonio-Santos J., da Silva Aragão R. (2024). Influence of maternal high-fat diet on offspring’s locomotor activity during anxiety-related behavioral tests: A systematic review. Behav. Brain Res..

[38] Chaves W.F., Pinheiro I.L., da Silva J.M., Manhães-de-Castro R., da Silva Aragão R. (2021). Repercussions of maternal exposure to high-fat diet on offspring feeding behavior and body composition: A systematic review. J. Dev. Orig. Health Dis..

[39] Almeida M.M., Dias-Rocha C.P., Souza A.S., Muros M.F., Mendonca L.S., Pazos-Moura C.C., Trevenzoli I.H. (2017). Perinatal maternal high-fat diet induces early obesity and sex-specific alterations of the endocannabinoid system in white and brown adipose tissue of weanling rat offspring. Br. J. Nutr..

[40] Yoon H., Shaw J.L., Haigis M.C., Greka A. (2021). Lipid metabolism in sickness and in health: Emerging regulators of lipotoxicity. Mol. Cell.

[41] Hirko K.A., Chai B., Spiegelman D., Campos H., Farvid M.S., Hankinson S.E., Willett W.C., Eliassen A.H. (2018). Erythrocyte membrane fatty acids breast cancer risk: A prospective analysis in the nurses’ health study II. Int. J. Cancer.

[42] Kitamura Y., Kogomori C., Hamano H., Maekawa I., Shimizu T., Shiga S. (2017). Relationship between Changes in Fatty Acid Composition of the Erythrocyte Membranes and Fatty Acid Intake during Pregnancy in Pregnant Japanese Women. Ann. Nutr. Metab..

[43] Létondor A., Buaud B., Vaysse C., Fonseca L., Herrouin C., Servat B., Layé S., Pallet V., Alfos S. (2014). Erythrocyte DHA level as a biomarker of DHA status in specific brain regions of *n*-3 long-chain PUFA-supplemented aged rats. Br. J. Nutr..

[44] Echeverría F., Ortiz M., Valenzuela R., Videla L.A. (2016). Long-chain polyunsaturated fatty acids regulation of PPARs, signaling: Relationship to tissue development and aging. Prostaglandins Leukot. Essent. Fatty Acids.

[45] McNamara R.K., Asch R.H., Lindquist D.M., Krikorian R. (2018). Role of polyunsaturated fatty acids in human brain structure and function across the lifespan: An update on neuroimaging findings. Prostaglandins Leukot. Essent. Fatty Acids.

[46] Zhao T., Huang H., Li J., Shen J., Zhou C., Xiao R., Ma W. (2023). Association between erythrocyte membrane fatty acids and gut bacteria in obesity-related cognitive dysfunction. AMB Express.

[47] Valenzuela R., Das U.N., Videla L.A., Llorente C.G. (2018). Nutrients and Diet: A Relationship between Oxidative Stress, Aging, Obesity, and Related Noncommunicable Diseases. Oxidative Med. Cell. Longev..

[48] Johnson M., Pace R.D., McElhenney W.H. (2018). Green leafy vegetables in diets with a 25:1 omega-6/omega-3 fatty acid ratio modify the erythrocyte fatty acid profile of spontaneously hypertensive rats. Lipids Health Dis..

[49] Ren Z., Okyere S.K., Xie L., Wen J., Wang J., Chen Z., Ni X., Deng J., Hu Y. (2022). Oral Administration of Bacillus toyonensis Strain SAU-20 Improves Insulin Resistance and Ameliorates Hepatic Steatosis in Type 2 Diabetic Mice. Front. Immunol..

[50] Ferré P., Phan F., Foufelle F. (2021). SREBP-1c and lipogenesis in the liver: An update. Biochem. J..

[51] Lee H.B., Do M.H., Jhun H., Ha S.K., Song H.S., Roh S.W., Chung W.-H., Nam Y.-D., Park H.-Y. (2021). Amelioration of Hepatic Steatosis in Mice through Bacteroides uniformis CBA7346-Mediated Regulation of High-Fat Diet-Induced Insulin Resistance and Lipogenesis. Nutrients.

[52] Liao T.C., Huang J.P., Tsai Y.T., Shih W.C., Juan C.C., Hsieh P.S., Hung L.-M., Yu C.-L. (2022). Granulocytic MDSC with Deficient CCR5 Alleviates Lipogenesis and Inflammation in Nonalcoholic Fatty Liver Disease. Int. J. Mol. Sci..

[53] Tsuduki T., Kitano Y., Honma T., Kijima R., Ikeda I. (2013). High Dietary Fat Intake During Lactation Promotes Development of Diet-Induced Obesity in Male Offspring of Mice. J. Nutr. Sci. Vitaminol..

[54] Thompson M.D., Cismowski M.J., Trask A.J., Lallier S.W., Graf A.E., Rogers L.K., Lucchesi P.A., Brigstock D.R. (2016). Enhanced Steatosis and Fibrosis in Liver of Adult Offspring Exposed to Maternal High-Fat Diet. Gene Expr..

[55] Scheidl T., Wager J.L., Baker L., Brightwell A., Melan K.M., Larion S., Sarr O., Regnault T.R., Urbanski S.J., Thompson J.A. (2023). High Maternal Adiposity During Pregnancy Programs an Imbalance in the Lipidome and Predisposes to Diet-Induced Hepatosteatosis in the Offspring. Biosci. Rep..

[56] Bautista C.J., Montaño S.A., Ramírez V.M., Escobedo-Morales A., Nathanielsz P.W., Bobadilla N.A., Zambrano E. (2015). Changes in Milk Composition in Obese Rats Consuming a High-Fat Diet. Br. J. Nutr..

[57] Treesukosol Y., Sun B., Moghadam A.A., Liang N.C., Tamashiro K.L.K., Moran T.H. (2014). Maternal High-Fat Diet During Pregnancy and Lactation Reduces the Appetitive Behavioral Component in Female Offspring Tested in a Brief-Access Taste Procedure. Am. J. Physiol. Regul. Integr. Comp. Physiol..

[58] Volpato A.M., Schultz A., Magalhães-da-Costa E., Correia M., Águila M.B., Mandarim-de-Lacerda C.A. (2012). Maternal High-Fat Diet Programs for Metabolic Disturbances in Offspring Despite Leptin Sensitivity. Neuroendocrinology.

[59] Sheldon R.D., Blaize A.N., Fletcher J.A., Donkin S.S., Newcomer S.C., Rector R.S. (2016). Gestational Exercise Protects Adult Male Offspring From High-Fat Diet-Induced Hepatic Steatosis. J. Hepatol..

[60] Urbonaite G., Knyzeliene A., Bunn F.S., Smalskys A., Neniskyte U. (2022). The impact of maternal high-fat diet on offspring neurodevelopment. Front. Neurosci..

[61] Dagnino-Subiabre A. (2021). Stress and Western diets increase vulnerability to neuropsychiatric disorders: A common mechanism. Nutr. Neurosci..

[62] de Kloet E.R., Joëls M. (2024). The cortisol switch between vulnerability and resilience. Mol. Psychiatry.

[63] McClafferty S.R., Paniagua-Ugarte C., Hannabass Z.M., Jackson P.A., Hayes D.M. (2024). Comparing the effects of infant maternal and sibling separation on adolescent behavior in rats (Rattus norvegicus). PLoS ONE.

