# Supplementary material for: High-Fat Diet in Perinatal Period Promotes Liver Steatosis and Low Desaturation Capacity of Polyunsaturated Fatty Acids in Dams: A Link with Anxiety-Like Behavior in Rats
Source: Nutrients. 2025 Mar 28;17(7):1180. doi: 10.3390/nu17071180 (PMC11990584; doi:10.3390/nu17071180)
Supplement: Supplementary file 1 [file nutrients-17-01180-s001.zip › nutrients-3539052-supplementary.pdf]

Table S1. Diets composition. information provided by Research diet, New Jersey, USA.

Product	D22072206 (HFD)		D22072209 (CD)	
	gm%	Kcal%	gm%	Kcal%
Protein	26	20	19	20
Carbohidrates	26	20	67	70
Lipids	35	60	4	10
Total		100		100
Kcal/gm	5,2		3,8	
Ingredients	gm	Kcal	gm	Kcal
Casein, 80 Mesh	200	800	200	800
L-Cistina	3	12	3	12
Corn starch	0	0	506,2	2024,8
Maltodextrin 10	125	500	125	500
Sucrose	68,8	275,2	68,8	275,2
Celulose, BW200	50	0	50	0
Hydrogenated coconut oil	100	900	10	90
Linseed oil	27,8	250	4	36
soybean oil	0	0	6	54
Safflower oil	66,4	598	5	45
Sunflower oil, high oleic	75,8	682	15	135
Lard	0	0	5	45
tBHQ	0	0	0	0
Mineral mix S10026	10	0	10	0
Dicalcium phosphate	13	0	13	0
Calcium carbonate	5,5	0	5,5	0
Potassium citrate 1 H20	16,5	0	16,5	0
Vitamin mix V10001	10	40	10	40
Choline bitartrate	2	0	2	0
Total	773,9	4057	1055,1	4057
LA (% of total lipids)		23,1		22,3
LA (g/kg Diet)		80,5		9,5
ALA (% of total lipids)		5,7		6,1
ALA (g/kg Diet)		20		2,6
Saturate (%)		41,9		32,1
Monoinsaturate (%)		28,9		38,8
Poliinsaturate (%)		29,2		29,1
n-6 PUFA		62,3		10
n-3 PUFA		15,5		2,7
n6:n3 PUFA ratio		4		3,7

Table S2. Gene specific TacMan probes used in the study.

Gen	Name	Gene Bank	Code	Product Lenght	Melting Tº
Ppara	Peroxisome proliferator activated receptor alpha	NM_013196.1	Rn00566193_m1	98	60ºC
Srebf1 (Srebp-1c)	Srebf 1 / Sterol regulatory element binding transcription factor 1	NM_001276707.1	Rn01495769_m1	79	60ºC
Acox1	ACOX 1 / Acyl-CoA oxidase 1, palmitoyl	NM_017340.2	Rn01460628_m1	63	60ºC
CPT1a	Carnitine palmitoyltransferase 1a	NM_031559.2	Rn00580702_m1	64	60ºC
Fasn	Fatty acid Synthase	NM_017332.1	Rn00569117_m1	74	60ºC
Acaca (Acc)	Acaca / ACC1 / Acetyl-CoA carbolxilase α	NM_022193.1	Rn00573474_m1	60	60ºC
Fads1 (Fas1)	Fads 1 / Fatty acid desaturase 1	NM_053445.2	Rn00584915_m1	86	60ºC
Fads2 (Fas2)	Fads 2 / Fadsd6 / Fatty acid desaturase 2	NM_031344.2	Rn00580220_m1	100	60ºC
Actb (Actina)	Actb	NM_031144.3	Rn00667869_m1	91	60ºC
GAPDH	Glyceraldehyde-3-phosphate dehydrogenase	NM_017008.4	Rn01775763_g1	174	60ºC

Table S3. Determination of fatty acids by gas chromatography in stomach content from offspring at PND7.

Fatty Acids (%mmol)	Stomach Content		
	CD (n=12)	HFD (n=12)	ANOVA p-value
**C14:0**	12.6 ± 2.1[a]	8.8 ± 0.5	**0,0196**
**C16:0**	31.1 ± 3.5[a]	15.5 ± 0.9	**<0,0001**
**C16:1**	3.9 ± 1.2[a]	0.4 ± 0.1	**0,0498**
**C18:0**	8.9 ± 4.2	7.7 ± 0.5	0,9994
**C18:1n9c**	33.4 ± 4.1	41.4 ± 1[a]	**<0,0001**
**C18:2n6c (LA)**	7.8 ± 1.5	23.9 ± 0.6[a]	**<0,0001**
**C18:3n3 (ALA)**	0.6 ± 0.1	0.5 ± 0.1	>0,9999
**C20:4n6 (AA)**	0.9 ± 0.2	1.1 ± 0.2	>0,9999
**C:22:5 n-6 (DPA)**	0.1 ± 0	0 ± 0	>0,9999
**C22:5 n-3 (DPA)**	0.2 ± 0.1	0.1 ± 0	>0,9999
**C20:5n3 (EPA)**	0.2 ± 0	0.2 ± 0	>0,9999
**C22:6n3 (DHA)**	0.4 ± 0.1	0.3 ± 0.1	>0,9999
**ΣSFA**	52.6 ± 6.6[a]	32.1 ± 1.5	**<0,0001**
**ΣMUFA**	37.3 ± 5.2	41.8 ± 1.1[a]	**0,0022**
**ΣPUFA**	10.1 ± 1.7	26.1 ± 0.5[a]	**<0,0001**
**Σn-6 PUFA**	8.7 ± 1.6	25.0 ± 0.5[a]	**<0,0001**
**Σn-3 PUFA**	1.4 ± 0.1	1.1 ± 0.1	>0,9999
**Ratio n6:n3**	6.2 ± 0.7	22.0 ± 2.0[a]	**<0,0001**

Sample consider 6 male and 6 female of each diet. Data presented as mean ± SD. SFA: Saturated fatty acids; MUFA: Monounsaturated fatty acids; PUFA: Polyunsaturated fatty acids. CD: Control diet and HFD: high fat diet. Differences were calculated by t test [a]$P < 0.05$ between CD and HFD.
